# Association between exposure to ambient air pollution and hospital admission, incidence, and mortality of stroke: an updated systematic review and meta-analysis of more than 23 million participants

**DOI:** 10.1186/s12199-021-00937-1

**Published:** 2021-01-26

**Authors:** Zhiping Niu, Feifei Liu, Hongmei Yu, Shaotang Wu, Hao Xiang

**Affiliations:** 1 Department of Global Health, School of Health Sciences, Wuhan University, 115# Donghu Road, Wuhan, China; 2 Global Health Institute, Wuhan University, 115# Donghu Road, Wuhan, China; 3 School of Management, Chengdu University of Traditional Chinese Medicine, 37# Shierqiao Road, Chengdu, China

**Keywords:** Stroke, Air pollution, Hospital admission, Incidence, Mortality

## Abstract

**Background:**

Previous studies have suggested that exposure to air pollution may increase stroke risk, but the results remain inconsistent. Evidence of more recent studies is highly warranted, especially gas air pollutants.

**Methods:**

We searched PubMed, Embase, and Web of Science to identify studies till February 2020 and conducted a meta-analysis on the association between air pollution (PM_2.5_, particulate matter with aerodynamic diameter less than 2.5 μm; PM_10_, particulate matter with aerodynamic diameter less than 10 μm; NO_2_, nitrogen dioxide; SO_2_, sulfur dioxide; CO, carbon monoxide; O_3_, ozone) and stroke (hospital admission, incidence, and mortality). Fixed- or random-effects model was used to calculate pooled odds ratios (OR)/hazard ratio (HR) and their 95% confidence intervals (CI) for a 10 μg/m^3^ increase in air pollutant concentration.

**Results:**

A total of 68 studies conducted from more than 23 million participants were included in our meta-analysis. Meta-analyses showed significant associations of all six air pollutants and stroke hospital admission (e.g., PM_2.5_: OR = 1.008 (95% CI 1.005, 1.011); NO_2_: OR = 1.023 (95% CI 1.015, 1.030), per 10 μg/m^3^ increases in air pollutant concentration). Exposure to PM_2.5_, SO_2_, and NO_2_ was associated with increased risks of stroke incidence (PM_2.5_: HR = 1.048 (95% CI 1.020, 1.076); SO_2_: HR = 1.002 (95% CI 1.000, 1.003); NO_2_: HR = 1.002 (95% CI 1.000, 1.003), respectively). However, no significant differences were found in associations of PM_10_, CO, O_3_, and stroke incidence. Except for CO and O_3_, we found that higher level of air pollution (PM_2.5_, PM_10_, SO_2_, and NO_2_) exposure was associated with higher stroke mortality (e.g., PM_10_: OR = 1.006 (95% CI 1.003, 1.010), SO_2_: OR = 1.006 (95% CI 1.005, 1.008).

**Conclusions:**

Exposure to air pollution was positively associated with an increased risk of stroke hospital admission (PM_2.5_, PM_10_, SO_2_, NO_2_, CO, and O_3_), incidence (PM_2.5_, SO_2_, and NO_2_), and mortality (PM_2.5_, PM_10_, SO_2_, and NO_2_). Our study would provide a more comprehensive evidence of air pollution and stroke, especially SO_2_ and NO_2_.

**Supplementary Information:**

The online version contains supplementary material available at 10.1186/s12199-021-00937-1.

## Introduction

Stroke, characterized by acute cerebral blood circulation disorder, is caused by artery stenosis, occlusion, or rupture caused by various inducing factors in patients with cerebrovascular diseases [1]. Stroke has become a leading contributor to the global burden of disease and the second leading cause of death worldwide [2, 3]. According to the Global Burden of Disease Study (GBD) report, there were approximately 80.1 million stroke patients, and 5.5 million deaths were attributed to stroke in 2016 globally [4]. Considering stroke is characterized with high incidence, high mortality, and contribute to severe burden disease, identifying potential risk factor of stroke is of great significance for public health. In parallel, air pollution has also been regarded as one of the major environmental problems and a risk factor of many cardiovascular diseases (CVD), including stroke [5]. GBD 2019 showed that air pollution was globally the sixth leading cause of stroke death during 1990 to 2017, and 28.1% disability-adjusted life years (DALYs) of stroke attribute to environmental factors exposure [6, 7].

Air pollution is the most significant environmental risk factor for all-cause mortality [8]. Increasing number of human epidemiologic studies has been conducted to assess the potential association between air pollution exposure and stroke admission, incidence, and mortality in recent years. However, the results were inconsistent, and the associations between exposure to air pollution and stroke have not been fully understood. Some studies reported positive association between air pollution exposure and stroke hospital admission/incidence/mortality, whereas others did not [4, 9–14]. For example, Huang et al. 2019 indicated that exposure to PM_2.5_ was associated with increased stroke incidence and the adjusted risk ratio (RR) was 1.130 (95%CI: 1.090, 82 1.170) for each increase of 10 μg/m^3^ in n PM_2.5 _concentration [4]. The adjusted risk ratio (RR) was 1.130 (95% CI 1.090, 1.170) for each increase of 10 μg/m^3^ in PM_2.5_ concentration, while Wing et al. suggested no association was found between PM_2.5_ exposure and stroke incidence (RR = 0.950, 95% CI 0.710, 1.280) [11]. Previous meta-analyses have explored the associations between air pollution exposure and stroke [15–19]. However, these studies were mainly focused on the studies of particulate matter (PM_2.5_, particulate matter with aerodynamic diameter less than 2.5 μm; PM_10_, particulate matter with aerodynamic diameter less than 10 μm) and stroke outcomes [16–19]; results of gas air pollutants (NO_2_, nitrogen dioxide; SO_2_, sulfur dioxide; CO, carbon monoxide; O_3_, ozone) were scarce. Moreover, to the best of our knowledge, more than 30 studies exploring the association between air pollution exposure and stroke, especially conducted from the multi-city level and with large sample sizes, were published after the most recent meta-analysis. The more recent and comprehensive studies should be included in the meta-analysis to conclude an updated pooled effect estimate.

We therefore conducted an updated systematic review and meta-analysis to assess the association between 6 main air pollutants (PM_2.5_, PM_10_, NO_2_, SO_2_, CO, and O_3_) and 3 stroke outcomes (hospital admission, incidence, and mortality). This systematic review and meta-analysis was performed according to the guidelines of the Preferred Reporting Items for Systematic Review and Meta-analyses (PRISMA) criteria (Table S1).

## Methods

### Search strategy

Literature was searched in three databases (PubMed, Embase, and Web of Science), with published date until 1 February 2020. The search strategy was pairwise of combinations of terms concerning air pollution (e.g., air pollution, particulate matter, particles, PM_2.5_, PM_10_, nitrogen oxides (NOx), NO_2_, SO_2_, CO, and O_3_) and stroke (e.g., stroke, cerebrovascular disease, cerebrovascular disorder, cerebral hemorrhage, cerebral infarction, subarachnoid hemorrhage).

We first selected articles by screening titles and abstracts and then the full texts of potentially eligible studies were further evaluated. Reference lists of all the included studies were also manually searched. Literature selection was finished by two independent authors (ZP N and FF L), and conflicts between the two authors were resolved by discussing with an arbitrator (H X).

### Inclusion and exclusion criteria

Articles that met the following criteria were included: (1) provided quantitative measure of the associations between air pollution exposure with stroke admission, incidence, and/or mortality (relative risk (RR), odds ratio (OR), or hazard ratio (HR), and their 95% confidence interval (95%CI); (2) cohort, cross-sectional, time series, cross-sectional, case-control, case-crossover, or panel studies; (3) focused on outdoor (ambient) air pollution exposure but not indoor air pollution; (4) original peer-reviewed human subject research studies; (5) published in English. Studies were excluded if they were (1) toxicological studies, summaries, or reviews, and (2) articles without effect estimates after contacting the authors. In addition, for more than one article conducted from the same population, only the most recent studies were included.

### Data extraction

Data were extracted from all eligible studies, including the following: (1) study characteristics (first author, published year, study location, and period); (2) study population (sample size, proportion of males, range of age, mean age); (3) outcome (type of stroke and outcome was admission, incidence, and/or mortality); (4) air pollution assessment method and increment of air pollution used in effect estimates (per interquartile range (IQR), standard deviation (SD), or per 10 μg/m^3^); (5) effect estimates of the association between air pollution and stroke risk (OR, RR, HR with 95% CI). The effect estimates of single-pollutant model, generally called “main model” or “fully adjusted model,” were extracted [20].

### Quality assessment

Two authors (ZP N and FF L) worked independently, and inconsistencies in quality assessment were resolved through discussion. We employed the Newcastle-Ottawa Scale (NOS) to evaluate the quality of included studies. The NOS Tool has designed 8 items to assess the critical appraisal of the potential risk of bias. Total score of NOS ranged from 0–9. Study score higher than or equal to 7 was regarded as high-quality; otherwise, the study was regarded as “low quality” [21].

### Statistical analyses

This meta-analysis focused on examining the association between air pollution and three stroke outcomes, including admission, incidence, and/or mortality. We extracted effect estimates (OR, HR, RR, and 95%CI) from individual studies and then converted them into a standardized form of per 10 μg/m^3^ increases in air pollution. The significance of the pooled OR, RR, or HR was determined by the *Z* test [22], and *p* value less than 0.05 was considered statistically significant. Standard error (SE) for each effect estimate was calculated by using the formula: (upper limit − lower limit)/3.92.

Heterogeneity among studies was evaluated using *I*^2^ statistics and *Q* test [23]. If the values of *I*^2^ > 50% or *p* < 0.01, the heterogeneity was “high” and random effect model was used to pool estimates. Otherwise, heterogeneity was considered as “low or moderate,” and fixed-effect model was used to pool estimates.

Begg’s test and Egger’s test were conducted to assess publication bias. The influence of individual studies on the pooled estimates was examined by removing each study from the analysis one by one. Moreover, we also performed sensitivity analysis and subgroup analysis to evaluate if the exposure period would change the significance of the pooled results. Because long-term studies were limited, sensitivity analysis was conducted by omitting long-term exposure (cohort) studies. Subgroup analysis was only performed if the number of short-term exposure studies or long-term exposure studies was more than 3. Publication bias and sensitivity analysis were only performed if the number of included studies was more than 5. All statistical analysis was performed in Stata version 15.0 (StataCorp, College Station, TX, USA).

## Results

### Literature search and characteristics of included studies

**Fig. 1:**
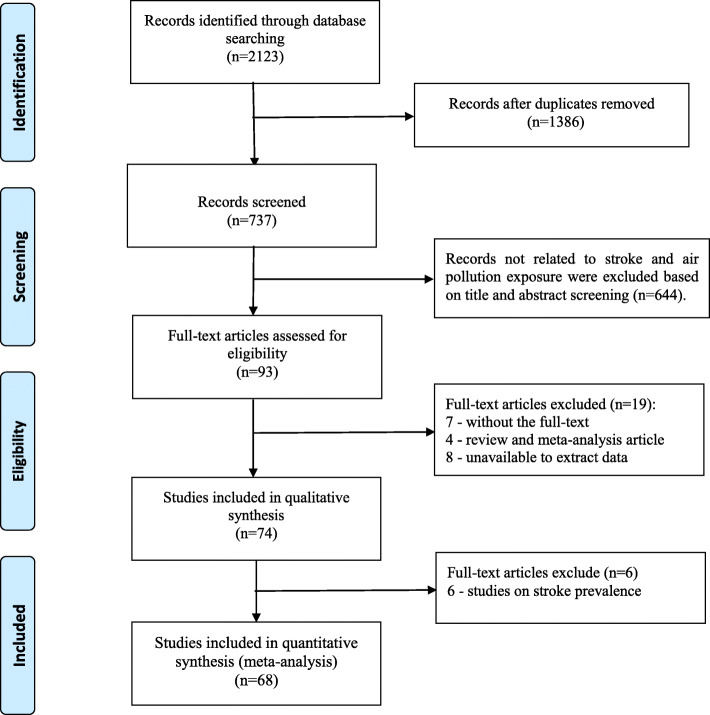
Flow chart of selecting studies for meta-analysis

After removing duplicates, 737 records were identified in the initial literature search. By reviewing title and abstracts, 93 studies were downloaded for full-text reading. According to the inclusion and exclusion criteria, a total of 68 studies were included in our meta-analysis (Fig. 1).

Table 1 provides the characteristics of 68 studies included in meta-analysis. As for air pollution involved in the study, there were 26 studies that reported the association between air pollution exposure and stroke hospital admission, 19 reported air pollution exposure and stroke incidence, 19 reported air pollution exposure and stroke mortality, and 3 reported both stroke incidence and mortality. The sample size of included studies ranged between 407 and 8,834,533; more than 23 million participants were included in meta-analysis eventually. Furthermore, the studies included were conducted from 18 countries. Time-series and cross-sectional were the most commonly adopted study designs. In our meta-analysis, all 68 included studies were considered as “high quality,” and the average NOS score was 8.26 for all studies (Table S2)

**Table 1:** Descriptive summaries for all included studies

Reference	Study Location and period	Study population	Study design	Exposure	Exposure assessment method	Type of stroke	Outcome
Huang et al. [4]	15 provinces in China, 2000–2015	117,575 Chinese men and women without stroke from the Atherosclerotic Cardiovascular Disease Risk in China (China-PAR) project	Cohort	PM_2.5_	A satellite-based spatiotemporal model, 1 × 1-km spatial resolution	All types of stroke	Incidence
Tian et al. [24]	172 cities in China, 2014–2016	2,032,667 hospital admissions for ischemic stroke in 172 cities in China	Time-series	PM_2.5_	1–17 monitors in each city operated by the National Air Pollution Monitoring System	Ischemic stroke	Hospital admission
Chen et al. [25]	Jinan, China, 2013–2015	56,922 stroke admissions	Case-crossover	PM_2.5_, PM_10_, SO_2_, NO_2_, O_3_	14 fix-sited monitoring stations in urban areas of Jinan operated by Jinan Environment Monitoring Center	All types of stroke	Hospital admission
Chen et al. [26]	China, 2007–2008	12,291 ischemic stroke patients from first national hospital-based prospective registry cohort of stroke in China	Cohort	PM_2.5_, PM_10_, NO_2_	Monitoring data, satellite remote sensing, meteorological and land use information	Ischemic stroke	Mortality
Xue et al. [27]	China, 2013–2015	1356 first-ever stroke events	Case-crossover	O_3_	1463 continuous air pollution monitoring sites operated by the China Environmental Protection Ministry	All types of stroke	Incidence
Qian et al. [28]	Shanghai, China, 2012–2014	5286 fatal intracerebral hemorrhage (ICH) case	Case-crossover	PM_2.5_	The Shanghai Environmental Monitoring Center	Hemorrhagic stroke	Incidence
Tian et al. [12]	184 major cities in China, 2014–2017	8,834,533 hospital admissions for cardiovascular causes in 184 Chinese cities	Time-series	PM_2.5_	The National Air Pollution Monitoring System	Ischemic, hemorrhagic stroke	Hospital admission
Tian et al. [10]	172 cities in China, 2014–2016	2,032,667 hospital admissions for ischemic stroke in 172 cities in China	Time-series	PM_2.5_, SO_2_, NO_2_, O_3_, CO	1–17 monitors in each city operated by the National Air Pollution Monitoring System	Ischemic stroke	Hospital admission
Dong et al. [1]	Changzhou, China, 2015–2016	32,840 ischemic stroke (IS) cases, 4028 IS deaths	Time-series	PM_2.5_, PM_10_, SO_2_, NO_2_, CO	10 air quality monitoring stations operated by the Changzhou Environmental Monitoring Center	Ischemic stroke	Mortality, incidence
Zhong et al. [29]	Changsha city, China, 2008–2009	1536 stroke patients	Case-crossover	PM_10_, NO_2_, SO_2_	the Changsha Municipal Public Weather Information Service Website	All types of stroke	Hospital admission
Vivanco-Hidalgo et al. [30]	Barcelona, Spain, 2005–2014	27,421,536 stroke patients	Time-series	PM_2.5_	An urban background research site located in southwest Barcelona	Ischemic stroke	Incidence
Yitshak-Sade et al. [31]	New England, 2001–2011	2,015,660 stroke admissions	Time-series	PM_2.5_	Monitor PM data and aerosol optic depth (AOD) values (1 × 1 km)	Ischemic stroke	Hospital admission
Liu et al. [32]	272 cities in China, 2013–2015	294,199 deaths due to stroke in 272 Chinese cities	Time-series	CO	The National Urban Air Quality Real-time Publishing Platform	All types of stroke	Mortality
Wang et al. [33]	6 subtropical cities in China, 2013–2016	54,236 stroke deaths from six Chinese subtropical cities	Case-crossover	PM_2.5_, PM_10_	Municipal air monitoring system	All types of stroke	Mortality
Collart et al. [34]	Wallonia, Belgium, 2008–2011,	113,147 hospital admissions due to stroke	Time-series	NO_2_	ISSeP (the Scientific Institute of Public Services)	All types of stroke	Hospital admission
Chen et al. [35]	30 counties in China, 2013–2015	49,669 stroke deaths	Time-series	PM_2.5_	Fixed-site monitoring station operated by the closest spatial distance to the county center. Daily air pollution data for PM2.5 and O3 concentrations were collected from the National Air pollution Monitoring System	All types of stroke	Mortality
Wang et al. [36]	272 cities in China, 2013–2015	294,199 deaths due to stroke in 272 Chinese cities	Time-series	SO_2_	The National Urban Air Quality Real-time Publishing Platform	All types of stroke	Mortality
Chen et al. [9]	272 cities in China, 2013–2015	294,199 deaths due to stroke in 272 Chinese cities	Time-series	PM_2.5_	The National Urban Air Quality Real-time Publishing Platform	All types of stroke	Mortality
Yin et al. [37]	272 cities in China, 2013–2015	294,199 deaths due to stroke in 272 Chinese cities	Time-series	O_3_	The National Urban Air Quality Real-time Publishing Platform	All types of stroke	Mortality
Ha et al. [38]	USA, 2002–2008	228,438 deliveries	Case-crossover	PM_2.5_, PM_10_, SO_2_, O_3_, CO	Community Multiscale Air Quality (CMAQ) models	All types of stroke	Incidence
Huang et al. [39]	Beijing, China, 2013–2014	147,624 stroke admissions	Case-crossover	SO_2_, NO_2_, O_3_, CO	The Centre of City Environmental Protection Monitoring Website Platform of Beijing	All types of stroke	Hospital admission
Guo et al. [5]	South China, 2013–2015	95,562 ischemic stroke cases	Time-series	PM_2.5_, NO_2_, SO_2_, O_3_, CO	The Qingyue Open Environmental Data (QOED) Center	Ischemic stroke	Hospital admission
Liu et al. [40]	14 large cities in China, 2014–2015	200,958 ischemic stroke and 41,746 hemorrhagic stroke hospitalizations	Case-crossover	PM_10_, SO_2_, O_3_, CO, O_3_	The National Air Pollution Monitoring System	Ischemic, hemorrhagic stroke	Hospital admission
Wing et al. [11]	Texas, USA, 2000–2012	3216 first-ever ischemic strokes	Case-crossover	PM_2.5_, O_3_	The Texas Commission on Environmental Quality’s Texas Air Monitoring Information System from a centrally located monitor	Ischemic stroke	Incidence
Liu et al. [41]	26 cities in China, 2014–2015	348,379 stroke admissions	Case-crossover	PM_2.5_, PM_10_	The National Air Pollution Monitoring System	Ischemic, hemorrhagic stroke	Hospital admission
McClure et al. [42]	USA, 2003–2011	30,239 participants in the Reasons for Geographic and Racial Differences in Stroke (REGARDS) study, 746 incidences	Case-crossover	PM_2.5_	Moderate Resolution Imaging Spectroradiometer instrument on the NASA Aqua satellite (10 km × 10 km)	All types of stroke	Incidence
Tian et al. [43]	Beijing, China, 2010–2012	63,956 first hospital admissions due to stroke	Case-crossover	PM_2.5_	An ambient air quality monitoring station on the rooftop of embassy building located in Chaoyang district, Beijing	Ischemic stroke	Hospital admission
Lin et al. [44]	6 low- and middle-income countries, 2007–2010	45,625 participants from the Study on Global Aging and Adult Health	Cohort	PM_2.5_	Global estimates of ambient fine particulate matter concentrations from satellite-based aerosol optical depth	All types of stroke	Incidence
Hong et al. [45]	Changzhou, China, 2015–2016	32,840 ischemic stroke (IS) cases, 4028 IS deaths	Time-series	O_3_	10 air quality monitoring stations operated by the Changzhou Environmental Monitoring Center	Ischemic stroke	Incidence, mortality
Stockfelt et al. [46]	Gothenburg, Sweden, 1990–2011	1391 cases of stroke from the Primary Prevention Study (PPS) cohort and GOT-MONICA cohort	Cohort	PM_2.5_, PM_10_	High-resolution dispersion modeling was performed for the period 1990–2011 over a Gothenburg region domain (93 × 112 km)	All types of stroke	Incidence
Qiu et al. [47]	Hong Kong, China, 1998–2010,	6,733 cases of incident stroke	Cohort	PM_2.5_	Satellite-based aerosol optical depth (AOD) recordings and monitoring data from ground-based stations	All types of stroke	Incidence
Crichton et al. [48]	South London, England, 2005–2012	1800 incidence due to stroke	Time-series	PM_2.5_, PM_10_, NO_2_, O_3_	The KCLurban model developed at King’s College London	Ischemic, hemorrhagic stroke	Incidence
Huang et al. [49]	Beijing, China, 2013–2014	147,624 stroke admissions	Case-crossover	PM_2.5_, PM_10_	The Centre of City Environmental Protection Monitoring Website Platform of Beijing	Ischemic, hemorrhagic stroke	Hospital admission
Lin et al. [50]	Guangzhou, China, 2007–2011	9066 stroke deaths	Time-series	PM_2.5_, PM_10_	An automatic air monitoring system was installed on the rooftop of Panyu Meteorological Centre	All types of stroke	Mortality
Han et al. [51]	South Korea, 2004–2014	1,477 consecutive hemorrhagic stroke events	Case-crossover	PM_10_, NO_2_, O_3_	The Climate and Air Quality Management Division of South Korea	Hemorrhagic stroke	Incidence
Montresor-López et al. [13]	South Carolina, USA, 2002–2006	21,301 stroke patients	Case-crossover	O_3_	The US Environmental Protection Agency (USEPA), Hierarchical Bayesian Model (HBM)	All types of stroke	Hospital admission
Korek et al. [52]	Stockholm, Sweden, 1991–2010	22,587 individuals in four cohorts	Cohort	PM_10_	The Airviro Air Quality Management System	All types of stroke	Incidence
Chang et al. [53]	Tropical City, Taiwan, 2006–2010	27,392 admissions due to stroke	Case-crossover	PM_2.5_	6 air quality monitoring stations in Kaohsiung city operated by the Taiwanese Environmental Protection Administration (EPA)	All types of stroke	Hospital admission
Tian et al. [54]	Hong Kong, 2004–2011	140,774 emergency hospital admissions	Time-series	CO	4 general monitoring stations operated by the Environmental Protection Department (EPD) of Hong Kong	All types of stroke	Hospital admission
To et al. [55]	Canada, 1998–2006	89,835 women of the Canadian National Breast Screening Study (CNBSS)	Cohort	PM_2.5_	Satellite-based estimates of surface concentrations of PM_2.5_	All types of stroke	Incidence
Hoffmann et al. [56]	German, 2008–2009	4433 subjects from the German Heinz Nixdorf Recall cohort	Cohort	PM_2.5_, PM_10_	Land-use regression (LUR) models	All types of stroke	Incidence
Chen et al. [57]	Taiwan, 2006–2010	27,392 hospital admissions due to stroke	Case-crossover	PM_2.5_, PM_10_	6 air quality monitoring stations established in Kaohsiung city operated by the Taiwanese Environmental Protection Administration (EPA)	All types of stroke	Hospital admission
Amancio and Nascimento [58]	Brazil, 2005–2009	1,032 deaths due to stroke	Time-series	PM_10_, SO_2_	A measuring station in downtown São José dos Campos	All types of stroke	Mortality
Chen et al. [59]	Taiwan, 2004–2008,	12,982 ischemic, 3362 hemorrhagic stroke cases	Time-series	PM_2.5_	The Sinjhuang Supersite located in the center of the Taipei metropolitan area	Hemorrhage, ischemic stroke	Hospital admission
Stafoggia et al. 2014 [60]	European, 2006–2010	99,446 study participants from 11 European Cohorts within the European Study of Cohorts for Air Pollution Effects (ESCAPE) Project	Cohort	PM_2.5,_ PM_10_, NO_2_	Land-use regression (LUR) models	All types of stroke	Incidence
Chiu et al. [61]	Taipei, Taiwan, 2006–2010	12,520 hemorrhagic stroke (HS) hospital admissions for the 47 hospitals	Case-crossover	PM_2.5_	Air quality monitoring stations operated by the Taiwanese Environmental Protection Administration (EPA)	Hemorrhagic stroke	Hospital admission
Chen et al. [62]	8 cities in China, 1996–2008	4820,000 subjects of 8 Chinese cities, approximately	Time-series	PM_10_, SO_2_, NO_2_	2–12 monitoring stations in each city operated by the Ministry of Environmental Protection of China	All types of stroke	Mortality
Carlsen et al. [63]	Reykjavík, Iceland, 2003–2009	24,439 emergency hospital admissions due to stroke	Time-series	PM_10_, NO_2_, O_3_	The Environmental Branch of the Municipality of Reykjavík (2003–2008) and the Icelandic Environmental Protection Agency (2009)	All types of stroke	Hospital admission
Johnson et al. [64]	Canada, 2007–2009	4,696 stroke (cases) and 37,723 injury patients (controls)	Case-crossover	NO_2_	Land-use regression (LUR) model for the city of Edmonton	All types of stroke	Hospital admission
Atkinson et al. [65]	England, 2003–2007	836,557 patients	Cohort	PM_10_, NO_2_, NO_2_, O_3_	Air dispersion models (1 × 1-km grids)	All types of stroke	Incidence
Xu et al. [66]	Pennsylvania, USA, 1994–2000	26,210 hospital admissions due to stroke	Case-crossover	O_3_	The repository of ambient air quality database of the US Environmental Protection Agency	All types of stroke	Hospital admission
Xiang et al. [67]	Wuhan, China, 2006–2008	10,663 stroke hospital admissions from 4 major hospitals	Case-crossover	PM_10_, SO_2_, NO_2_	9 fixed-site stations operated by the Wuhan Environmental Monitoring Center	All types of stroke	Hospital admission
Yorifuji et al. [68]	Shizuoka, Japan, 1999–2009	14,001 elderly residents	Cohort	NO_2_	Land use regression (LUR) model	Hemorrhage, ischemic stroke	Mortality
Qian et al. [69]	Shanghai, China, 2003–2008	66,366 stroke deaths for adults aged over 65	Case-crossover	PM_10_, SO_2_, NO_2_	6 fixed-site stations operated by Shanghai Environmental Monitoring Center	All types of stroke	Mortality
Andersen et al [70]	Denmark, 1971–2006	52,215 participants of the Danish Diet, Cancer and Health cohort	Cohort	NO_2_	The Danish geographic information system-based air pollution and human exposure modeling system	All types of stroke	Incidence, Mortality
Nascimento et al. [71]	São Paulo State, Brazil, 2007–2008,	407 hospitalizations due to stroke	Time-series	PM_10_, SO_2_, O_3_	Measuring station of the São Paulo State Environmental Agency	All types of stroke	Hospital admission
OʼDonnell et al. [72]	Canada, 2003–2008,	9202 patients hospitalized due to ischemic stroke	Case-crossover	PM_2.5_	19 monitoring stations in the vicinity of the 11 regional stroke centers participating in the Registry	Ischemic Stroke	Incidence
Lipsett et al. [73]	California, USA, 1996–2005,	124,614 women living in California	Cohort	PM_2.5_, PM_10_, SO_2_, NO_2_, CO, O_3_	Fixed-site monitors, inverse distance weighting (IDW) interpolation	All types of stroke	Incidence
Yorifuji et al. [68]	Tokyo, Japan, 2003–2008	41,440 deaths due to stroke	Time-series	PM_2.5_, NO_2_	2 monitoring stations in Tokyo’s 23 wards	Hemorrhagic stroke	Mortality
Ren et al. [74]	Massachusetts, USA, 1995–2002	157,197 non-accident deaths aging 35 years or older	Case-crossover	O_3_	The Environmental Protection Agency, USA	All types of stroke	Mortality
Zanobetti and Schwartz [75]	USA, 1999–2005	330,613 deaths for stroke in 112 US cities	Time-series	PM_2.5_	Air Quality System Technology Transfer Network	All types of stroke	Mortality
Kettunen et al. [76]	Helsinki, Finland, 1998–2004	3265 deaths due to stroke	Time-series	PM_2.5_, PM_10_, NO_2_, CO, O_3_	The Environmental Protection Agency, USA	All types of stroke	Mortality
Franklin et al. [77]	USA,1997–2002	1310,781 deaths in 27 US communities	Case-crossover	PM_2.5_	National, State, and Local Ambient Monitoring Stations	All types of stroke	Mortality
Qian et al. [78]	Wuhan, China, 2001–2004	89,131 non-accidental death cases	Time-series	PM_10_	Wuhan Environmental Monitoring Center	All types of stroke	Mortality
Villeneuve et al. [79]	Edmonton, Canada, 1992–2002	12,422 stroke visits	Time-series	PM_2.5_, PM_10_, SO_2_, NO_2_, CO, O_3_	Fixed-site monitoring stations maintained by Environment Canada	All types of stroke	Hospital admission
Henrotin et al. [80]	Dijon, France, 1994–2004	1487 patients with ischemic stroke and 220 patients with hemorrhagic stroke	Case-crossover	PM_10,_ SO_2_, CO, O_3_	The monitoring station located in the town center, Dijon	Hemorrhage, ischemic stroke	Incidence
Tsai et al. [81]	Kaohsiung, Taiwan, 1997–2000	23,179 hospital admissions due to stroke	Case-crossover	PM_10,_ SO_2_, NO_2_, CO, O_3_	6 air-quality monitoring stations operated by the Environmental Protection Administration (EPA)	All types of stroke	Hospital admission
Yu et al. [14]	Seoul, Korea, 1991–1997	7137 ischemic deaths due to stroke	Time-series	SO_2_, NO_2_, CO, O_3_	20 monitoring site and data operated by the Department of the Environment (Seoul)	Ischemic stroke	Mortality

*PM*_*2.5*_ particulate matter with aerodynamic diameter less than 2.5 μm, *PM*_*10*_ particulate matter with aerodynamic diameter less than 10 μm, *SO*_*2*_ sulfur dioxide, *NO*_*2*_ nitrogen dioxide, *CO* carbon monoxide, *O*_*3*_ ozone, *China-PAR* Atherosclerotic Cardiovascular Disease Risk in China (China-PAR) project, *ICH* intracerebral hemorrhage, *IS* ischemic stroke, *REGARDS Cohort* Reasons for Geographic and Racial Differences in Stroke Cohort, *PPS* primary prevention study, *CNBSS* Canadian National Breast Screening Study, *HS* hemorrhagic stroke, *ESCAPE Project* the European Study of Cohorts for Air Pollution Effects, *AOD* aerosol optic depth, *ISSeP* the Scientific Institute of Public Services, *CMAQ* Community Multiscale Air Quality model, *QOED* the Qingyue Open Environmental Data Center, *USEPA* the US Environmental Protection Agency, *HBM* Hierarchical Bayesian Model, *EPA* the Taiwanese Environmental Protection Administration, *EPD* the Environmental Protection Department, *LUR* land use regression model, *IDW* inverse distance weighting

.

### Air pollution and stroke hospital admission

A total of 29 studies were performed to assess the association for air pollution and stroke hospital admission, and the results were inconsistent. Most studies showed a positive correlation between exposure to air pollution and the risk of hospital admission for stroke. In meta-analysis, we enrolled 13 studies on PM_2.5_, 11 studies on PM_10_ and NO_2_, 10 studies on SO_2_ and O_3_, and 6 studies on CO with stroke hospital admission and suggested an increased stroke hospital admission risk after air pollution exposure. The pooled odds ratio (OR) of stroke with a 10 μg/m^3^ increase in PM_2.5_, PM_10_, SO_2_, NO_2_, CO, and O_3_ was 1.008 (95% CI 1.005, 1.011), 1.004 (95% CI 1.001, 1.006), 1.013 (95% CI 1.007, 1.020), 1.023 (95% CI 1.015, 1.030), 1.000 (95% CI 1.000, 1.001), and 1.002 (95% CI 1.000, 1.003), respectively (Table 2, Figure S1-S6). Heterogeneity among studies was significant (*I*^2^ ≥ 50%, *p* < 0.001).

**Table 2:** Association between exposure to air pollution and stroke hospital admission (per 10 μg/m^3^ increment)

Air pollution	Hospital admission		Heterogeneity	
	NO.	HR (95% CI)	*I*^2^ (%)	*P*
PM_2.5_	19	1.008 (1.005, 1.011)	96.6	0.000
PM_10_	15	1.004 (1.001, 1.006)	92.7	0.000
SO_2_	13	1.013 (1.007, 1.020)	94.5	0.000
NO_2_	15	1.023 (1.015, 1.030)	92.6	0.000
CO	8	1.000 (1.000, 1.001)	92.7	0.000
O_3_	15	1.002 (1.000, 1.003)	80.2	0.000

*HR* hazard ratio, *NO.* number, *PM*_*2.5*_ particulate matter with aerodynamic diameter less than 2.5 μm, *PM*_*10*_ particulate matter with aerodynamic diameter less than 10 μm, *SO*_*2*_ sulfur dioxide, *NO*_*2*_ nitrogen dioxide, *CO* carbon monoxide, *O*_*3*_ ozone

### Air pollution and stroke incidence

Twenty-three studies have investigated the association of air pollution on stroke incidence (Table 1). For meta-analysis, we extracted 18 studies on PM_2.5_, 13 studies on PM_10_, 10 studies on O_3_, 7 studies on NO_2_, and 4 studies on SO_2_ and CO. Ten of these studies suggested increased risks for stroke incidence for at least one of the investigated pollutants. Meta-analysis showed that exposure to PM_2.5_, SO_2_, and NO_2_ was associated with increased risks of stroke incidence, and the pooled HR with a 10 μg/m^3^ increase was 1.048 (95% CI 1.020, 1.076), 1.002 (95% CI 1.000, 1.003), 1.002 (95% CI 1.000, 1.003), respectively. However, no significant differences were found in associations of PM_10_, CO, O_3_, and stroke incidence (Table 3, Figure S7-S12).

**Table 3:** Association between exposure to air pollution and stroke incidence (per 10 μg/m^3^ increment)

Air pollution	Incidence		Heterogeneity	
	NO.	OR (95% CI)	*I*^2^ (%)	*P*
PM_2.5_	18	1.048 (1.020, 1.076)	82.3	0.000
PM_10_	13	1.017 (0.981, 1.055)	51.9	0.010
SO_2_	4	1.002 (1.000, 1.003)	20.3	0.288
NO_2_	7	1.002 (1.000, 1.003)	0.0	0.512
CO	5	0.999 (0.997, 1.001)	0.0	0.763
O_3_	10	0.999 (0.999, 1.000)	34.1	0.135

*OR* odds ratios, *NO.* number, *PM*_*2.5*_ particulate matter with aerodynamic diameter less than 2.5 μm, *PM*_*10*_ particulate matter with aerodynamic diameter less than 10 μm, *SO*_*2*_ sulfur dioxide, *NO*_*2*_ nitrogen dioxide, *CO* carbon monoxide, *O*_*3*_ ozone

### Air pollution and stroke mortality

Twenty-two population-based studies have explored the association for exposure to air pollution and stroke mortality. As for meta-analysis, 11 articles on PM_2.5_, 10 articles on NO_2_, 9 articles on PM_10_, 6 articles on O_3_, and 4 articles on CO exposure were included. Meta-analysis showed that exposure to ambient PM_2.5_ (OR = 1.008 95% CI 1.005, 1.012, per 10 μg/m^3^ increment), PM_10_ (OR = 1.006, 95% CI 1.003, 1.010, per 10 μg/m^3^ increment), SO_2_ (OR = 1.006, 95% CI 1.005, 1.008, per 10 μg/m^3^ increment), and NO_2_ (OR = 1.009, 95% CI 1.003, 1.016, per 10 μg/m^3^ increment) was associated with increased risks of mortality due to stroke. No significant difference was shown in association between CO, O_3_ exposure, and stroke mortality (Table 4, Figure S13-S18).

**Table 4:** Association between exposure to air pollution and stroke mortality (per 10 μg/m^3^ increment)

Air pollution	Mortality		Heterogeneity	
	NO.	OR (95% CI)	*I*^2^ (%)	*P*
PM_2.5_	12	1.008 (1.005, 1.012)	89.2	0.000
PM_10_	10	1.006 (1.003, 1.010)	83.3	0.000
SO_2_	6	1.006 (1.005, 1.008)	45.8	0.100
NO_2_	10	1.009(1.003, 1.016)	70.1	0.000
CO	5	1.045 (0.980, 1.115)	50.6	0.091
O_3_	6	1.005 (0.999, 1.010)	84.8	0.000

*OR* odds ratios, *NO.* number, *PM*_*2.5*_ particulate matter with aerodynamic diameter less than 2.5 μm, *PM*_*10*_ particulate matter with aerodynamic diameter less than 10 μm, *SO*_*2*_ sulfur dioxide, *NO*_*2*_ nitrogen dioxide, *CO* carbon monoxide, *O*_*3*_ ozone

### Publication bias and sensitivity analysis

Publication bias of studies on PM_10_ exposure and stroke hospital admission may exist, since *p* values of Begg’s test were less than 0.05. Publication bias of studies was remarkable in association of exposure to PM_2.5_ and O_3_ and stroke incidence according to funnel plots and Egger’s test. For PM_2.5_ and stroke mortality, the *p* value of Egger’s test was 0.009, suggesting publication bias may exist. Other publication bias test indicated that no substantial publication bias of studies was observed according to funnel plots, Begg’s test, and Egger’s test (Table S3, Figure S19-34).

Sensitivity analysis showed that the relation of exposure to CO and stroke hospital admission might be influenced by Tian et al.’s study [10]. And the association between exposure to NO_2_ and stroke incidence may be influenced by Dong et al.’s study [1]. The pooled OR of exposure to air pollution and stroke mortality might be influenced by some studies (PM_2.5_: Wang et al.’s study [33]; O_3_: Yin et al.’s study [37]). We recalculated the pooled OR/HR and 95% CI after removing those studies (Table S3). Due to limited studies after excluding those studies, the pooled estimated effects of SO_2_ and stroke incidence and O_3_ and stroke mortality were not recalculated. Other sensitivity analyses indicated that excluding each individual study did not change the results, suggesting the results of the meta-analysis were stable (Table S4, Figure S35-50). Sensitivity analyses by exposure period found that the pooled effect estimates were not changed significantly after excluding the long-term (cohort) studies (Table S5). Subgroup analysis suggested that both short-term and long-term exposure to air pollution would increase the risk of stroke incidence (PM_2.5_, PM_10_, and NO_2_) and mortality (NO_2_) (Table S6).

## Discussion

We conducted a systematic review and meta-analysis of 68 epidemiological studies and performed a comprehensive evaluation on exposure ambient air pollution and stroke, which were conducted from more than 23 million participants. Most studies suggested that exposure to a higher level of air pollution was associated with increased stroke risk. Meta-analysis showed that exposures to air pollutants were associated with increased risk of stroke hospital admission (PM_2.5_, PM_10_, SO_2_, NO_2,_ CO, and O_3_), incidence (PM_2.5_, SO_2_, and NO_2_), and mortality (PM_2.5_, PM_10_, SO_2_, and NO_2_). Although the high heterogeneity may reduce the credibility of the pooled evidence to some extent, the large number of studies included and the consistency of the results indicated that our conclusions were credible to some extent.

The positive associations between exposure to PM_2.5_, PM_10_, SO_2_, NO_2_, CO, and O_3,_ and stroke hospital admission were observed in other meta-analysis. Yang et al. meta-analyzed 34 case-crossover and time series studies and reported significant associations for PM_10_ (per 10 μg/m^3^ increment: RR = 1.007, 95% CI 1.001, 1.013) and O_3_ (per 10 ppb increment: RR = 1.036, 95% CI 1.016, 1.056), but non-significant association for PM_2.5_, SO_2_, NO_2_, and CO [15]. The meta-analysis performed by Yang et al. was not consistent with our current study completely, which might be caused by the different number of the included studies. To our knowledge, more than 16 studies have been published after 2014, and studies included in Yang et al.’s study were mainly conducted in Europe and North America. Data from more recent studies, especially low- and middle-income countries were not considered. Moreover, many studies conducted from the multi-city level and with large sample sizes have been published in recent years, which were more likely to find a significant association between air pollution and stroke hospital admission. For example, Tian et al. performed a time-series of more than 2 million hospital admissions for ischemic stroke in 172 cities in China and suggested that elevated incidence of ischemic stroke hospital admissions was associated with exposure to higher level of PM_2.5_ (RR = 1.003, 95% CI 1.002, 1.005, per 10 μg/m^3^ increment), SO_2_ (RR = 1.013, 95% CI 1.011, 1.017, per 10 μg/m^3^ increment), and NO_2_ (RR = 1.018, 95% CI 1.015, 1.022, per 10 μg/m^3^ increment) [24].

Three meta-analyses were conducted to examine the association between exposure to particulate matter (PM_2.5_ and PM_10_) and stroke incidence, whereas no meta-analysis of gas air pollutants was published before the current study. Li et al. performed a meta-analysis to explore the association between PM_10_ and stroke incidence in time-series studies and case-crossover studies. These studies indicated that PM_10_ was not associated with stroke incidence in the time-series design (HR = 1.002, 95% CI 0.999, 1.005, per 10 μg/m^3^ increment), but significantly associated in case-crossover studies (HR = 1.028, 95% CI 1.001, 1.057, per 10 μg/m^3^ increment). Meanwhile, PM_2.5_ exposure was related to an increased risk of stroke incidence in time-series design (HR = 1.006, 95% CI 1.002, 1.010, per 10 μg/m^3^ increment), but no significant association in case-crossover studies (HR = 1.016, 95% CI 0.937, 1.097, per 10 μg/m^3^ increment) [16]. Only 12 studies published before 2010 were included in Li et al.’s study. We updated the literature search up to 2020, which generated more than 10 studies. Moreover, Li et al. separately analyzed the data from time-series and case-crossover studies, which would reduce the number of studies calculated the pooled estimates. These might explain the inconsistency in between our study and Li et al.’s study. Yu et al. updated the literature search before 2012 and identified 19 studies [19]. Yu et al. found that exposure to PM_10_ was associated with an increased risk of stroke incidence (HR = 1.004, 95% CI 1.001, 1.008, per 10 μg/m^3^ increment), but exposure to PM_2.5_ was not significantly associated with stroke incidence (HR = 0.999, 95% CI 0.994, 1.003, per 10 μg/m^3^ increment) [19]. The results of these published meta-analyses were not exactly the same as our study, which might be due to more than 15 studies published after Yu et al.’s study. Moreover, we conducted a meta-analysis of gas air pollutants and stroke incidence and found that exposure to a higher level of SO_2_ and NO_2_ was associated with higher risk of stroke incidence, which may fill the gap of meta-analysis of gas air pollutants and stroke incidence. We also found that compared to short-term exposure, long-term exposure to air pollution may be associated with a higher risk of stroke incidence (PM_2.5_, PM_10_, and NO_2_), which may be explained by different pathophysiological pathways.

Studies investigating the association between exposure to air pollution and stroke mortality have been partly analyzed in two meta-analysis [15, 17]. Yang et al. evaluated the association between all 6 pollutants and suggested that stroke mortality increased 1.34% (95% CI 0.27, 2.42) per 10 μg/m^3^ increase in PM_2.5_, 0.65% (95% CI 0.54, 0.77) per 10 μg/m^3^ increase in PM_10_, 2.45% (95% CI 1.83, 3.07) per 10 parts per billion (ppb) increase in SO_2_, 7.78% (95% CI 4.49, 11.60) per 1 ppm increase in CO, and 1.50% (95% CI 0.37, 2.63) per 10 ppb increase in NO_2_, respectively [15]. Consistent with Yang et al.’s study, our meta-analysis also indicated that exposure to a higher level of PM_2.5_, PM_10_, SO_2_, and NO_2_ was related to higher risk of stroke mortality. No association was observed in both our study and Yang et al.’s study. However, Yang et al. reported a positive association in CO, whereas our study did not, which may be explained by the limited number of included studies. Scheers et al. performed a meta-analysis of exposure to PM_10_ and stroke events (mortality and incidence) and suggested that exposure to PM_10_ was positively associated with overall stroke events (mortality and incidence) (HR = 1.061, 95% CI 1.018, 1.105), but no significant association were observed in stroke mortality (HR = 1.080, 95% CI 0.992, 1.177) [17]. Inconsistency of Scheers et al.’s study and current study could be explained that Scheers et al.’s study included the studied estimated exposure to PM_10_ from studies using PM_2.5_, which may cause estimation bias to some extent.

Although accurate mechanisms of air pollution exposure and stroke remain unclear, several pathways including systemic inflammation, oxidative stress, thrombosis, and vascular endothelial dysfunction have been proposed [1, 9, 15, 82]. Vascular function injury may be central to mechanisms for air pollution-related stroke, which could lead to raised level of blood pressure and plasma viscosity [26]. It has been showed that exposure to air pollution was associated with increased thrombosis and vascular endothelial dysfunction by provoking oxidative stress and releasing systemic inflammatory cytokines [83]. Moreover, evidence also suggested that exposure to air pollution can lead to dysfunction of the autonomic system, which has been found as the major pathway that could result in air pollution-related adverse cardiovascular outcomes, such as stroke [84]. In addition, stroke status may aggravate the susceptibility of population to air pollution and increase the adverse cardiovascular effects of air pollution circularly [62].

A major strength of our meta-analysis is that our systematic review and meta-analysis covered six main air pollutants (PM_2.5_, PM_10,_ NO_2_, SO_2,_ CO, O_3_) and a rich set of stroke outcomes (hospital admission, incidence, and mortality), which may be difficult to obtain from individual studies or isolated reviews or meta-analyses. However, some limitations should be acknowledged. Firstly, high heterogeneity existed in some meta-analysis, which may be due to different study designs, difference in exposure assessment method and population demographics, and the varied covariable adjustment strategies in different studies. Secondly, our study failed to perform the association between different subtypes of stroke (ischemic stroke, hemorrhagic stroke) and air pollution exposure separately because most included studies (48 out of 68 articles) did not report subtypes of stroke or results of ischemic stroke and hemorrhagic stroke specifically. Finally, the correlation between different air pollutants was not examined in our study because different air pollutants were controlled in different studies, and the results of those studies could not be pooled directly.

## Conclusion

Our study demonstrated that exposure to air pollution was positively associated with an increased risk of stroke hospital admission (PM_2.5_, PM_10_, SO_2_, NO_2_, CO, and O_3_), incidence (PM_2.5_, SO_2_, and NO_2_), and mortality (PM_2.5_, PM_10_, SO_2_, and NO_2_). Given the great global burden of stroke and air pollution, our findings could provide some scientific evidence to accurate prevention and treatment of stroke and air pollution exposure.

## Supplementary Information

**Additional file 1.**

## Data Availability

All data described, analyzed, or discussed in this review are included in cited publications.

## Abbreviations

PM_2.5_: Particulate matter with aerodynamic diameter less than 2.5 μm
PM_10_: Particulate matter with aerodynamic diameter less than 10 μm
NO_2_: Nitrogen dioxide
SO_2_: Sulfur dioxide
CO: Carbon monoxide
O_3_: Ozone
OR: Odds ratio
HR: Hazard ratio
CI: Confidence interval
e.g.: For example
GBD: The Global Burden of Disease Study
CVD: Cardiovascular diseases
DALYs: Disability-adjusted life years
RR: Risk ratio
PRISMA: The Preferred Reporting Items for Systematic Review and Meta-analyses
NOx: Nitrogen oxides
IQR: Interquartile range
SD: Standard deviation
NOS: The Newcastle-Ottawa Scale
SE: Standard error
China-PAR: Atherosclerotic Cardiovascular Disease Risk in China (China-PAR) project
ICH: Intracerebral hemorrhage
IS: Ischemic stroke
REGARDS cohort: Reasons for Geographic and Racial Differences in Stroke Cohort
PPS: Primary prevention study
CNBSS: Canadian National Breast Screening Study
HS: Hemorrhagic stroke
ESCAPE Project: The European Study of Cohorts for Air Pollution Effects
AOD: Aerosol optic depth
ISSeP: The Scientific Institute of Public Services
CMAQ: Community Multiscale Air Quality model
QOED: The Qingyue Open Environmental Data Center
USEPA: The US Environmental Protection Agency
HBM: Hierarchical Bayesian Model
EPA: The Taiwanese Environmental Protection Administration
EPD: The Environmental Protection Department
LUR: Land-use regression model
IDW: Inverse distance weighting
