# Supplementary material for: Association between exposure to ambient air pollution and hospital admission, incidence, and mortality of stroke: an updated systematic review and meta-analysis of more than 23 million participants
Source: Environ Health Prev Med. 2021 Jan 26;26:15. doi: 10.1186/s12199-021-00937-1 (PMC7839211; doi:10.1186/s12199-021-00937-1)
Supplement: Supplementary file 1 — Additional file 1. [file 12199_2021_937_MOESM1_ESM.docx]

Supplementary materials

**Table S1.** PRISMA 2009 Checklist

| **Section/topic** | **#** | **Checklist item** | **Reported on page #** | |
| --- | --- | --- | --- | --- |
| **TITLE** | | |  | |
| Title | 1 | Identify the report as a systematic review, meta-analysis, or both. | Page 1 | |
| **ABSTRACT** | | |  | |
| Structured summary | 2 | Provide a structured summary including, as applicable: background; objectives; data sources; study eligibility criteria, participants, and interventions; study appraisal and synthesis methods; results; limitations; conclusions and implications of key findings; systematic review registration number. | Page 2,3 | |
| **INTRODUCTION** | | |  | |
| Rationale | 3 | Describe the rationale for the review in the context of what is already known. | Page 4,5 | |
| Objectives | 4 | Provide an explicit statement of questions being addressed with reference to participants, interventions, comparisons, outcomes, and study design (PICOS). | Page 5 | |
| **METHODS** | | |  | |
| Protocol and registration | 5 | Indicate if a review protocol exists, if and where it can be accessed (e.g., Web address), and, if available, provide registration information including registration number. | NA | |
| Eligibility criteria | 6 | Specify study characteristics (e.g., PICOS, length of follow-up) and report characteristics (e.g., years considered, language, publication status) used as criteria for eligibility, giving rationale. | Page 6,7 | |
| Information sources | 7 | Describe all information sources (e.g., databases with dates of coverage, contact with study authors to identify additional studies) in the search and date last searched. | Page 6,7 | |
| Search | 8 | Present full electronic search strategy for at least one database, including any limits used, such that it could be repeated. | Page 6 | |
| Study selection | 9 | State the process for selecting studies (i.e., screening, eligibility, included in systematic review, and, if applicable, included in the meta-analysis). | Page 6 | |
| Data collection process | 10 | Describe method of data extraction from reports (e.g., piloted forms, independently, in duplicate) and any processes for obtaining and confirming data from investigators. | Page 7 | |
| Data items | 11 | List and define all variables for which data were sought (e.g., PICOS, funding sources) and any assumptions and simplifications made. | Page 7 | |
| Risk of bias in individual studies | 12 | Describe methods used for assessing risk of bias of individual studies (including specification of whether this was done at the study or outcome level), and how this information is to be used in any data synthesis. | Page 8,9 | |
| Summary measures | 13 | State the principal summary measures (e.g., risk ratio, difference in means). | Page 8 | |
| Synthesis of results | 14 | Describe the methods of handling data and combining results of studies, if done, including measures of consistency (e.g., I2) for each meta-analysis. | Page 6 | |
| **Section/topic** | **#** | **Checklist item** | **Reported on page #** |  |
| Risk of bias across studies | 15 | Specify any assessment of risk of bias that may affect the cumulative evidence (e.g., publication bias, selective reporting within studies). | Page 8 |  |
| Additional analyses | 16 | Describe methods of additional analyses (e.g., sensitivity or subgroup analyses, meta-regression), if done, indicating which were pre-specified. | Page 8 |  |
| **RESULTS** | | |  |  |
| Study selection | 17 | Give numbers of studies screened, assessed for eligibility, and included in the review, with reasons for exclusions at each stage, ideally with a flow diagram. | Page 9 |  |
| Study characteristics | 18 | For each study, present characteristics for which data were extracted (e.g., study size, PICOS, follow-up period) and provide the citations. | Page 9, 27-30 |  |
| Risk of bias within studies | 19 | Present data on risk of bias of each study and, if available, any outcome level assessment (see item 12). | Page 11,12 |  |
| Results of individual studies | 20 | For all outcomes considered (benefits or harms), present, for each study: (a) simple summary data for each intervention group (b) effect estimates and confidence intervals, ideally with a forest plot. | Page 9-11, 31,32 |  |
| Synthesis of results | 21 | Present results of each meta-analysis done, including confidence intervals and measures of consistency. | Page 9-11, 32-33 |  |
| Risk of bias across studies | 22 | Present results of any assessment of risk of bias across studies (see Item 15). | Page 11,12 |  |
| Additional analysis | 23 | Give results of additional analyses, if done (e.g., sensitivity or subgroup analyses, meta-regression [see Item 16]). | Page 11,12 |  |
| **DISCUSSION** | | |  |  |
| Summary of evidence | 24 | Summarize the main findings including the strength of evidence for each main outcome; consider their relevance to key groups (e.g., healthcare providers, users, and policy makers). | Page 12,13 |  |
| Limitations | 25 | Discuss limitations at study and outcome level (e.g., risk of bias), and at review-level (e.g., incomplete retrieval of identified research, reporting bias). | Page 16,17 |  |
| Conclusions | 26 | Provide a general interpretation of the results in the context of other evidence, and implications for future research. | Page 17 |  |
| **FUNDING** | | |  |  |
| Funding | 27 | Describe sources of funding for the systematic review and other support (e.g., supply of data); role of funders for the systematic review. | Page 18 |  |

*From:*  Moher D, Liberati A, Tetzlaff J, Altman DG, The PRISMA Group (2009). Preferred Reporting Items for Systematic Reviews and Meta-Analyses: The PRISMA Statement. PLoS Med 6(7): e1000097. doi:10.1371/journal.pmed1000097

For more information, visit: **www.prisma-statement.org**.

| **Table S2.** Quality assessment of included studies | | | | |
| --- | --- | --- | --- | --- |
| Reference | Quality assessment (NOS) | | | Total score |
|  | Selection | Comparability | Outcome |  |
| Huang et al. 2019 | 4 | 2 | 2 | 8 |
| Tian et al. 2019 | 4 | 1 | 2 | 7 |
| Chen et al. 2019a | 4 | 1 | 3 | 8 |
| Chen et al. 2019b | 4 | 2 | 3 | 9 |
| Xue et al. 2019 | 4 | 2 | 3 | 9 |
| Qian et al. 2019 | 4 | 1 | 3 | 8 |
| Tian et al. 2019a | 4 | 2 | 2 | 8 |
| Tian et al. 2018 | 3 | 2 | 3 | 8 |
| Dong et al. 2018 | 4 | 1 | 2 | 7 |
| Zhong et al. 2018 | 4 | 2 | 3 | 9 |
| Vivanco-Hidalgo et al. 2018 | 4 | 1 | 3 | 8 |
| Yitshak-Sade et al. 2018 | 4 | 1 | 3 | 8 |
| Liu et al. 2018 | 3 | 2 | 3 | 8 |
| Wang et al. 2018 | 4 | 2 | 2 | 8 |
| Collart et al. 2018 | 4 | 2 | 3 | 9 |
| Chen et al. 2018 | 4 | 2 | 3 | 9 |
| Chen et al. 2017 | 3 | 2 | 3 | 8 |
| Yin et al. 2017 | 4 | 2 | 3 | 9 |
| Ha et al. 2017 | 4 | 2 | 2 | 8 |
| Huang et al. 2017 | 4 | 2 | 3 | 9 |
| Guo et al. 2017 | 4 | 2 | 3 | 9 |
| Liu et al.2017a | 4 | 2 | 3 | 9 |
| Wing et al. 2017 | 4 | 1 | 3 | 8 |
| Liu et al. 2017 | 4 | 1 | 3 | 8 |
| McClure et al. 2017 | 4 | 2 | 3 | 9 |
| Tian et al. 2017 | 4 | 2 | 3 | 9 |
| Lin et al. 2017 | 4 | 1 | 3 | 8 |
| Yu et al. 2017 | 4 | 2 | 3 | 9 |
| Stockfelt et al. 2017 | 4 | 2 | 3 | 9 |
| Qiu et al. 2017 | 4 | 2 | 3 | 9 |
| Crichton et al. 2016 | 4 | 2 | 2 | 8 |
| Huang et al. 2016 | 3 | 2 | 2 | 7 |
| Lin et al. 2016 | 4 | 1 | 3 | 8 |
| Han et al. 2016 | 4 | 2 | 3 | 9 |
| Montresor-López et al. 2016 | 4 | 2 | 3 | 9 |
| Korek et al. 2015 | 4 | 2 | 2 | 8 |
| Chang et al. 2015 | 4 | 2 | 3 | 9 |
| Tian et al. 2015 | 4 | 2 | 3 | 9 |
| To et al. 2015 | 4 | 2 | 3 | 9 |
| Hoffmann et al. 2015 | 3 | 2 | 3 | 8 |
| Chen et al. 2015 | 3 | 2 | 2 | 7 |
| Amancio et al. 2014 | 4 | 2 | 3 | 9 |
| Chen et al. 2014 | 4 | 1 | 3 | 8 |
| Stafoggia et al. 2014 | 4 | 1 | 3 | 8 |
| Chiu et al. 2014 | 4 | 2 | 3 | 9 |
| Chen et al. 2013 | 4 | 2 | 3 | 9 |
| Chen et al. 2013 | 4 | 2 | 3 | 9 |
| Carlsen et al. 2013 | 4 | 2 | 3 | 9 |
| Johnson et al. 2013 | 4 | 2 | 3 | 9 |
| Atkinson et al. 2013 | 4 | 2 | 3 | 9 |
| Xu et al. 2013 | 3 | 2 | 2 | 7 |
| Xiang et al. 2013 | 4 | 2 | 3 | 9 |
| Yorifuji et al. 2013 | 4 | 2 | 3 | 9 |
| Qian et al. 2013 | 4 | 1 | 3 | 8 |
| Andersen et al. 2012 | 4 | 1 | 3 | 8 |
| Nascimento et al. 2012 | 4 | 2 | 3 | 9 |
| OʼDonnell et al. 2011 | 4 | 2 | 3 | 9 |
| Lipsett et al. 2011 | 4 | 2 | 3 | 9 |
| Yorifuji et al. 2011 | 4 | 2 | 3 | 9 |
| Ren et al. 2010 | 4 | 2 | 3 | 9 |
| Zanobetti and Schwartz. 2009 | 4 | 2 | 3 | 9 |
| Kettunen et al. 2007 | 4 | 2 | 2 | 8 |
| Franklin et al. 2007 | 4 | 2 | 3 | 9 |
| Qian et al. 2007 | 3 | 1 | 3 | 7 |
| Villeneuve et al. 2006 | 4 | 1 | 3 | 8 |
| Tsai et al.2003 | 3 | 2 | 3 | 8 |
| Hong et al. 2002 | 3 | 1 | 3 | 7 |

| **Table S3**. Results of publication bias analysis | | | | | | | | | | | |
| --- | --- | --- | --- | --- | --- | --- | --- | --- | --- | --- | --- |
| Air pollution | Hospital Admission | | |  | Incidence | | |  | Mortality | | |
|  | NO. | Begg’s test | Egger’s test |  | NO. | Begg’s test | Egger’s test |  | NO. | Begg’s test | Egger’s test |
| PM_2.5_ | 19 | 0.080 | 0.172 |  | 18 | 0.834 | 0.011 |  | 12 | 0.304 | 0.009 |
| PM_10_ | 15 | 0.013 | 0.085 |  | 13 | 0.381 | 0.117 |  | 10 | 0.210 | 0.107 |
| SO_2_ | 13 | 0.161 | 0.644 |  | 4 | - | - |  | 6 | 0.452 | 0.111 |
| NO_2_ | 15 | 0.166 | 0.704 |  | 7 | 1.000 | 0.548 |  | 11 | 0.755 | 0.014 |
| CO | 8 | 1.000 | 0.268 |  | 5 | 0.462 | 0.183 |  | 5 | - | - |
| O_3_ | 15 | 0.350 | 0.131 |  | 10 | 0.876 | 0.027 |  | 6 | 0.707 | 0.114 |

Publication bias analysis and sensitivity analysis were only performed if the number of included was more than 5. PM_2.5_, particulate matter with aerodynamic diameter less than 2.5 µm; PM_10_, particulate matter with aerodynamic diameter less than 10 µm; SO_2_, sulfur dioxide; NO_2_, nitrogen dioxide; CO, carbon monoxide; O_3_, ozone.

| **Table S4.** Sensitivity analysis of the association between exposure to air pollution and stroke after removing influenced studies | | | | | | |
| --- | --- | --- | --- | --- | --- | --- |
| Air pollutants - stroke outcomes | Before removing influenced studies | |  | After removing influenced studies | |  |
|  | NO. | OR |  | NO. | OR |  |
| CO - hospital admission | 8 | 1.000 (1.000, 1.001) | 7 | | 1.001 (1.000, 1.001) |  |
| NO_2_- incidence | 8 | 1.002 (1.000, 1.003) | 7 | | 1.012 (0.990, 1.035) |  |
| PM_2.5_ - mortality | 12 | 1.008 (1.005, 1.012) | 11 | | 1.004 (1.001, 1.006) |  |

| **Table S5.** Sensitivity analysis of the association between exposure to air pollution and stroke by omitting long-term (cohort) studies | | | | | |
| --- | --- | --- | --- | --- | --- |
| Air pollutants - stroke outcomes | Before removing long-term (cohort) studies | |  | After removing long-term (cohort) studies | |
|  | NO. | OR |  | NO. | OR |
| PM_2.5_ - hospital admission | 19 | 1.008 (1.005, 1.011) |  | 19 | - |
| PM_10_ - hospital admission | 15 | 1.004 (1.001, 1.006) |  | 15 | - |
| SO_2_ - hospital admission | 13 | 1.013 (1.007, 1.020) |  | 13 | - |
| NO_2_ - hospital admission | 15 | 1.004 (1.001, 1.006) |  | 15 | - |
| CO - hospital admission | 8 | 1.000 (1.000, 1.001) |  | 8 | - |
| O_3_ - hospital admission | 15 | 1.023 (1.015, 1.030) |  | 15 | - |
| PM_2.5_ - incidence | 18 | 1.048 (1.020, 1.076) |  | 13 | 1.038 (1.007, 1.071) |
| PM_10_ - incidence | 13 | 1.019 (0.981, 1.058) |  | 7 | 1.019 (0.981, 1.057) |
| SO_2_ - incidence | 4 | 1.002 (1.000, 1.003) |  | 2 | 1.002 (1.000, 1.004) |
| NO_2_ - incidence | 7 | 1.002 (1.000, 1.003) |  | 3 | 1.002 (1.000, 1.003) |
| CO - incidence | 5 | 0.999 (0.997, 1.000) |  | 4 | 0.999 (0.999, 1.000) |
| O_3_ - incidence | 10 | 0.999 (0.999, 1.000) |  | 7 | 0.999 (0.999, 1.000) |
| PM_2.5_ -mortality | 12 | 1.010 (1.006, 1.014) |  | 10 | 1.009 (1.005, 1.013) |
| PM_10_ - mortality | 10 | 1.006 (1.003, 1.010) |  | 9 | 1.006 (1.003, 1.010) |
| SO_2_ - mortality | 6 | 1.006 (1.005, 1.008) |  | 6 | - |
| NO_2_ - mortality | 11 | 1.008(1.002, 1.014) |  | 6 | 1.006 (1.000,1.012) |
| CO - mortality | 5 | 1.045(0.980, 1.115) |  | 5 | - |
| O_3_ - mortality | 6 | 1.005(0.999, 1.010) |  | 6 | - |

- Sensitivity analysis was not performed because there were no long-term (cohort) studies included.

| **Table S6.** Subgroup analysis of short-term exposure studies or long-term exposure studies | | | | |
| --- | --- | --- | --- | --- |
| Air pollutants - stroke outcomes | Subgroup | NO. | OR | Weight |
| PM_2.5_ - incidence | Overall | 18 | 1.048 (1.020, 1.076) | 100.00% |
|  | Short-term exposure | 14 | 1.038 (1.007, 1.071） | 64.75% |
|  | Long-term exposure | 4 | 1.081 (0.971, 1.023） | 35.25% |
| PM_10_ - incidence | Overall | 13 | 1.017 (0.981, 1.055) | 100.00% |
|  | Short-term exposure | 8 | 1.019 (0.981, 1.057) | 70.56% |
|  | Long-term exposure | 5 | 1.033 (0.907, 1.175) | 29.44% |
| NO_2_ – incidence | Overall | 7 | 1.002 (1.000, 1.003） | 100.00% |
|  | Short-term exposure | 4 | 1.002 (1.000, 1.003) | 99.75% |
|  | Long-term exposure | 3 | 1.005 (0.977, 1.034) | 0.25% |
| NO_2_ - mortality | Overall | 11 | 1.008 (1.002, 1.014) | 100.00% |
|  | Short-term exposure | 6 | 1.006 (1.000, 1.012) | 73.35% |
|  | Long-term exposure | 5 | 1.047 (0.995, 1.101) | 26.65% |
| - Subgroup analysis were only performed if the number of short-term exposure studies or long-term exposure studies was more than 3. | | | | |


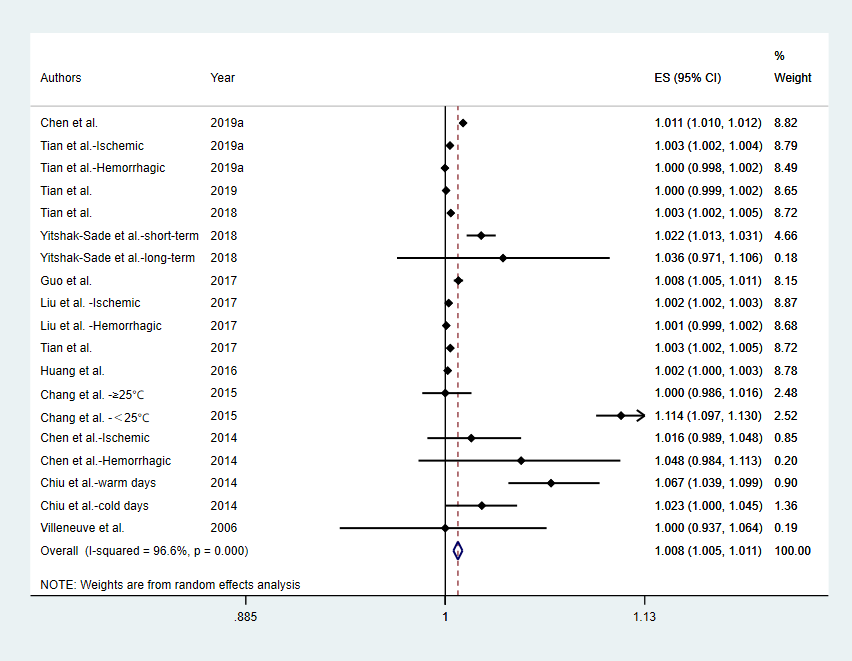

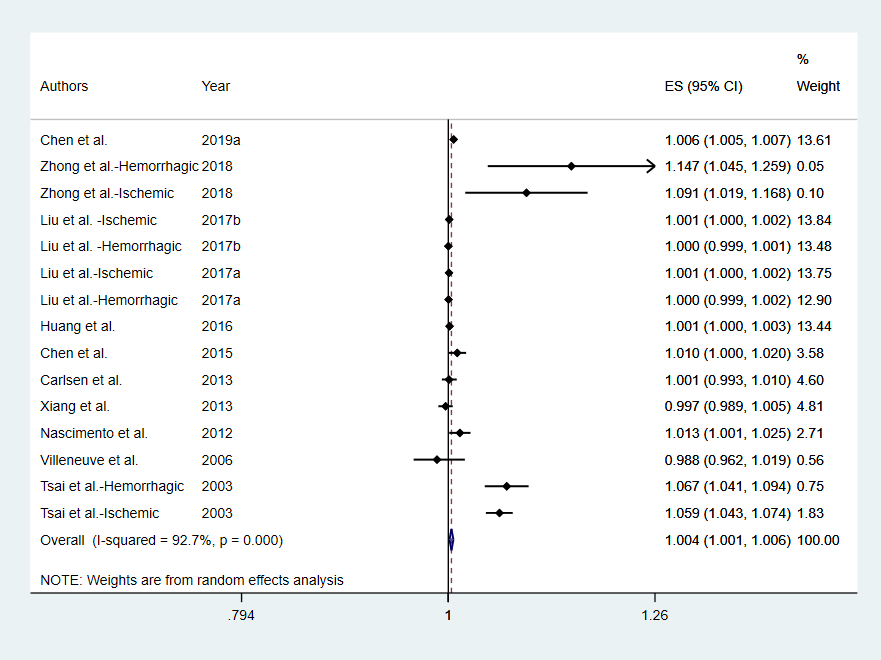
**Figure S1**. Meta-analysis of stroke hospital admission and exposure to PM_2.5_ for increments of 10 μg/m^3^.

**Figure S2**. Meta-analysis of stroke hospital admission and exposure to PM_10_ for increments of 10 μg/m^3^.


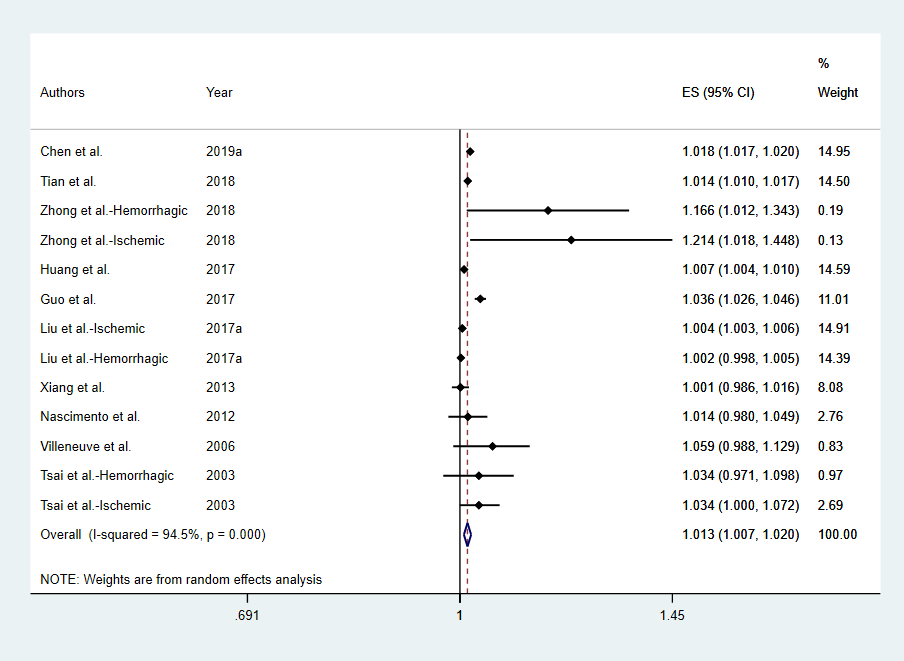


**Figure S3**. Meta-analysis of stroke hospital admission and exposure to SO_2_ for increments of 10 μg/m^3^.


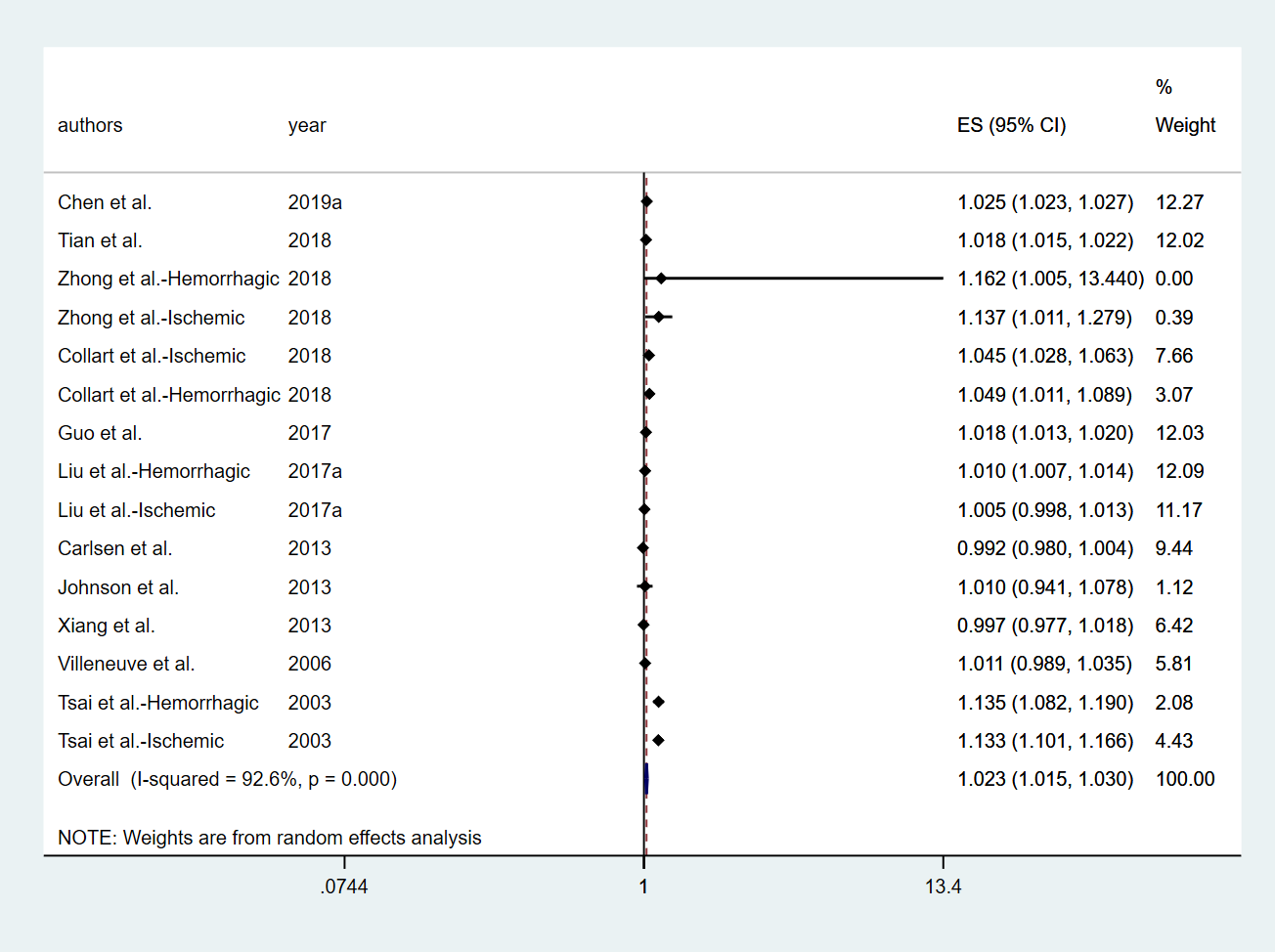


**Figure S4**. Meta-analysis of stroke hospital admission and exposure to NO_2_ for increments of 10 μg/m^3^.


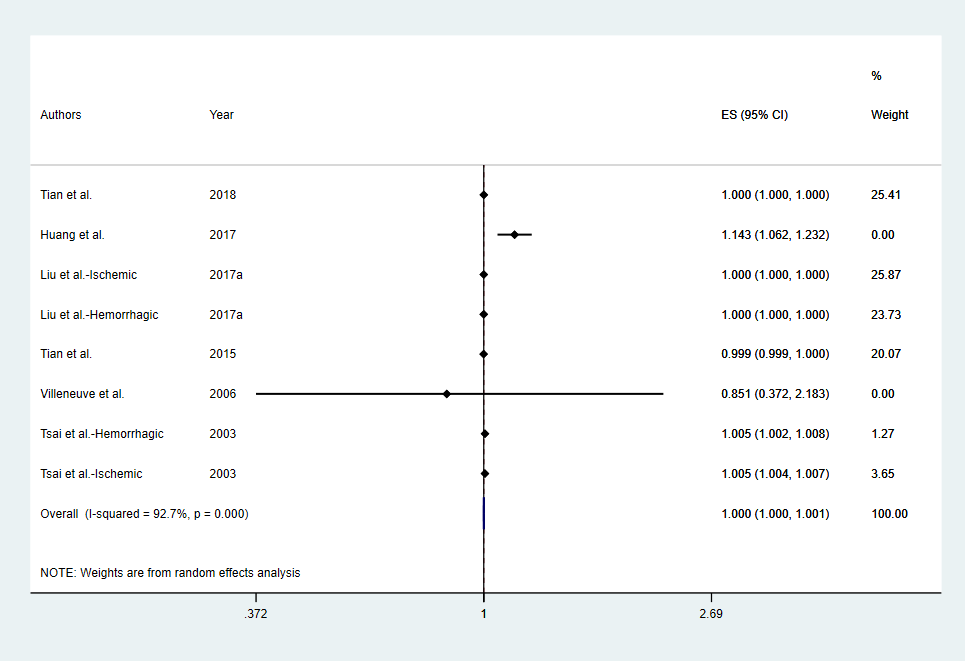


**Figure S5**. Meta-analysis of stroke hospital admission and exposure to CO for increments of 10 μg/m^3^.


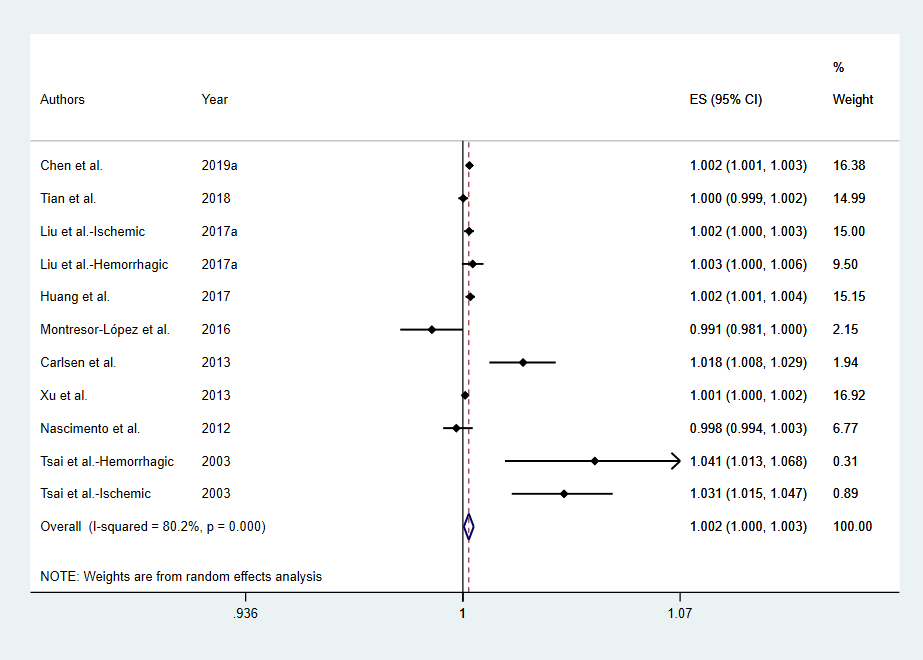


**Figure S6**. Meta-analysis of stroke hospital admission and exposure to O_3_ for increments of 10 μg/m^3^.


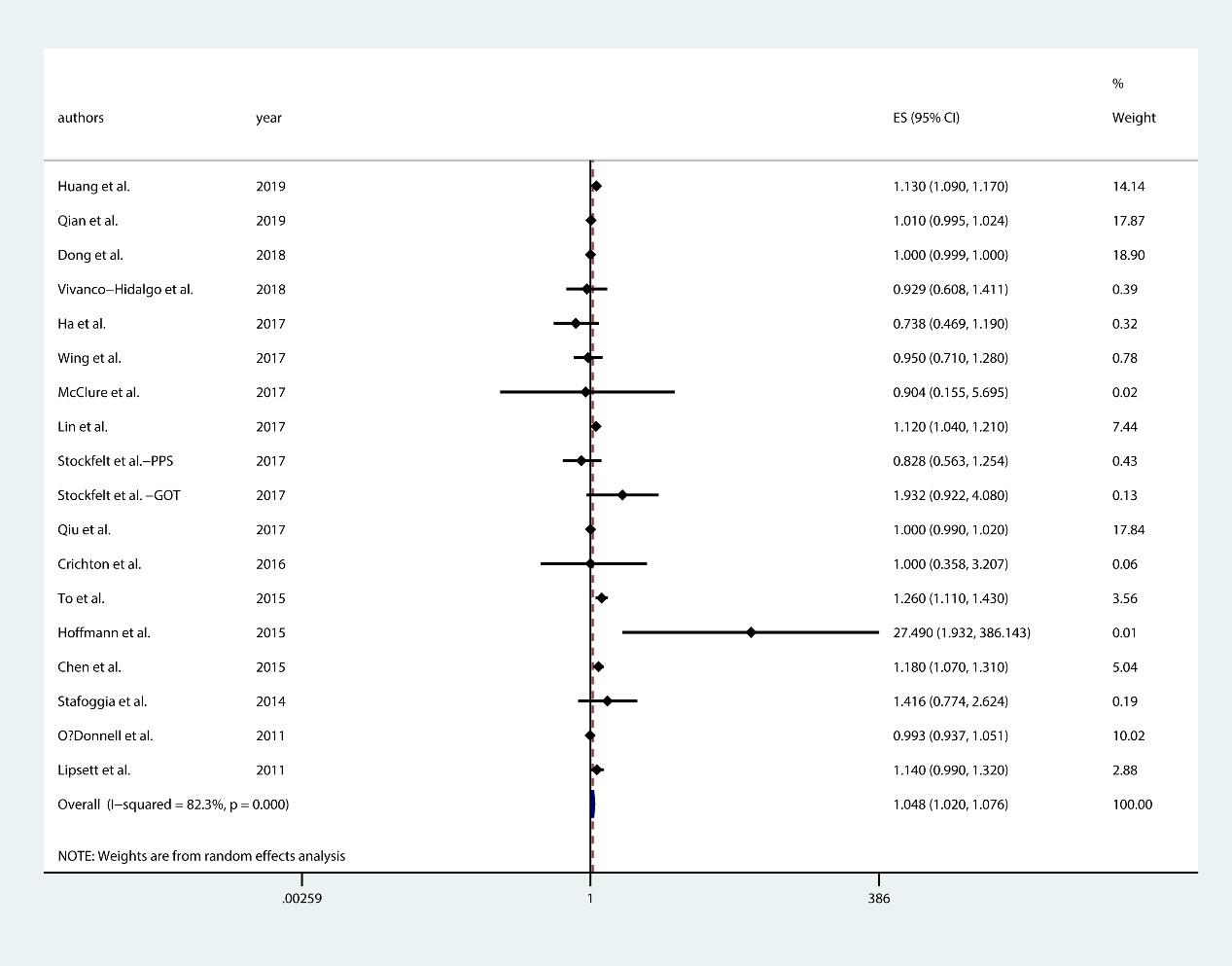


**Figure S7**. Meta-analysis of stroke incidence and exposure to PM_2.5_ for increments of 10 μg/m^3^.


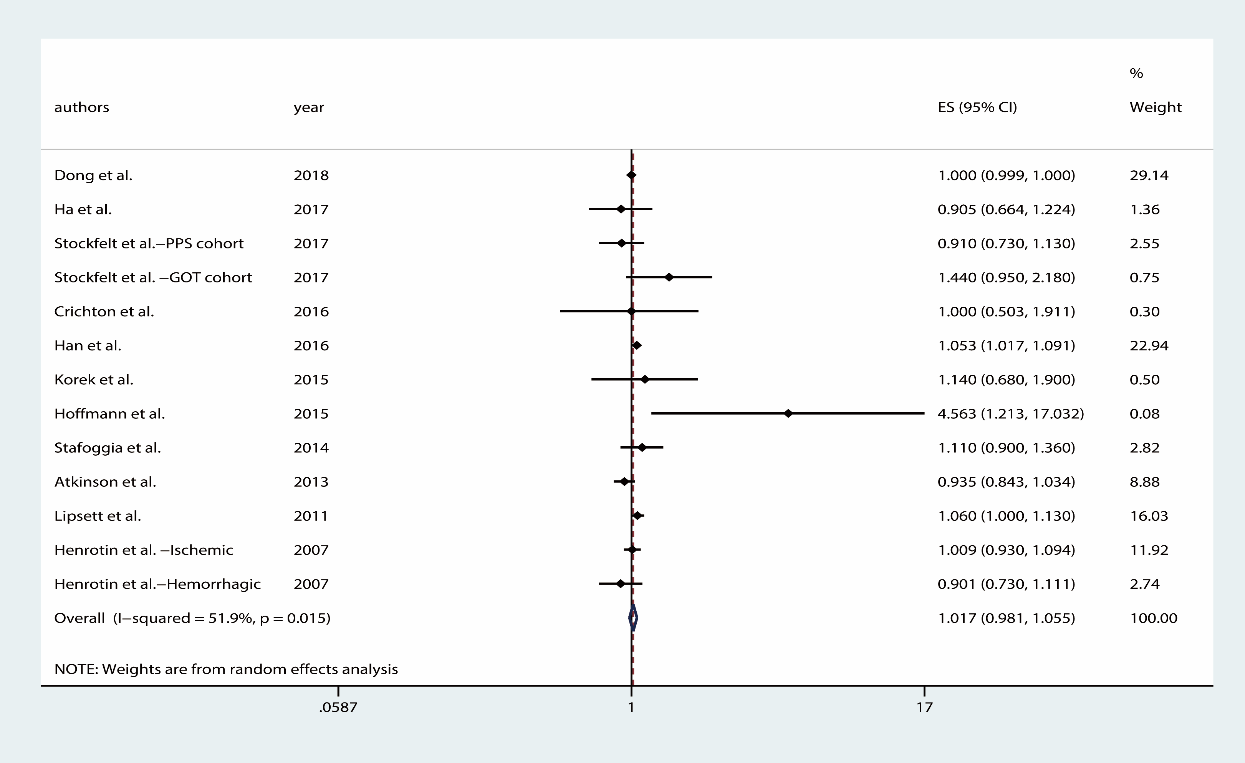


**Figure S8**. Meta-analysis of stroke incidence and exposure to PM_10_ for increments of 10 μg/m^3^.


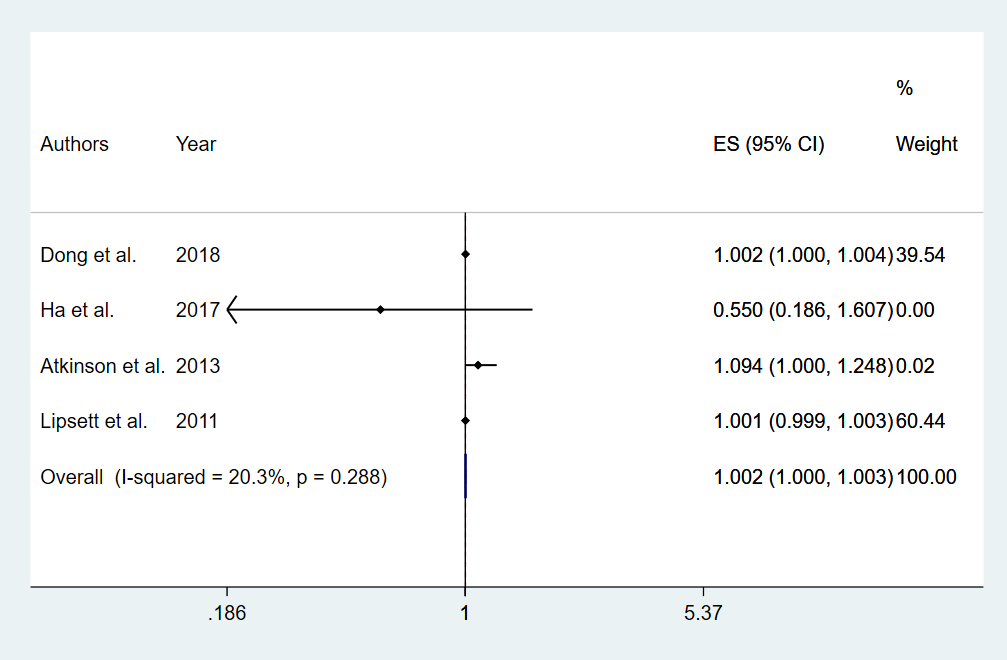


**Figure S9**. Meta-analysis of stroke incidence and exposure to SO_2_ for increments of 10 μg/m^3^.


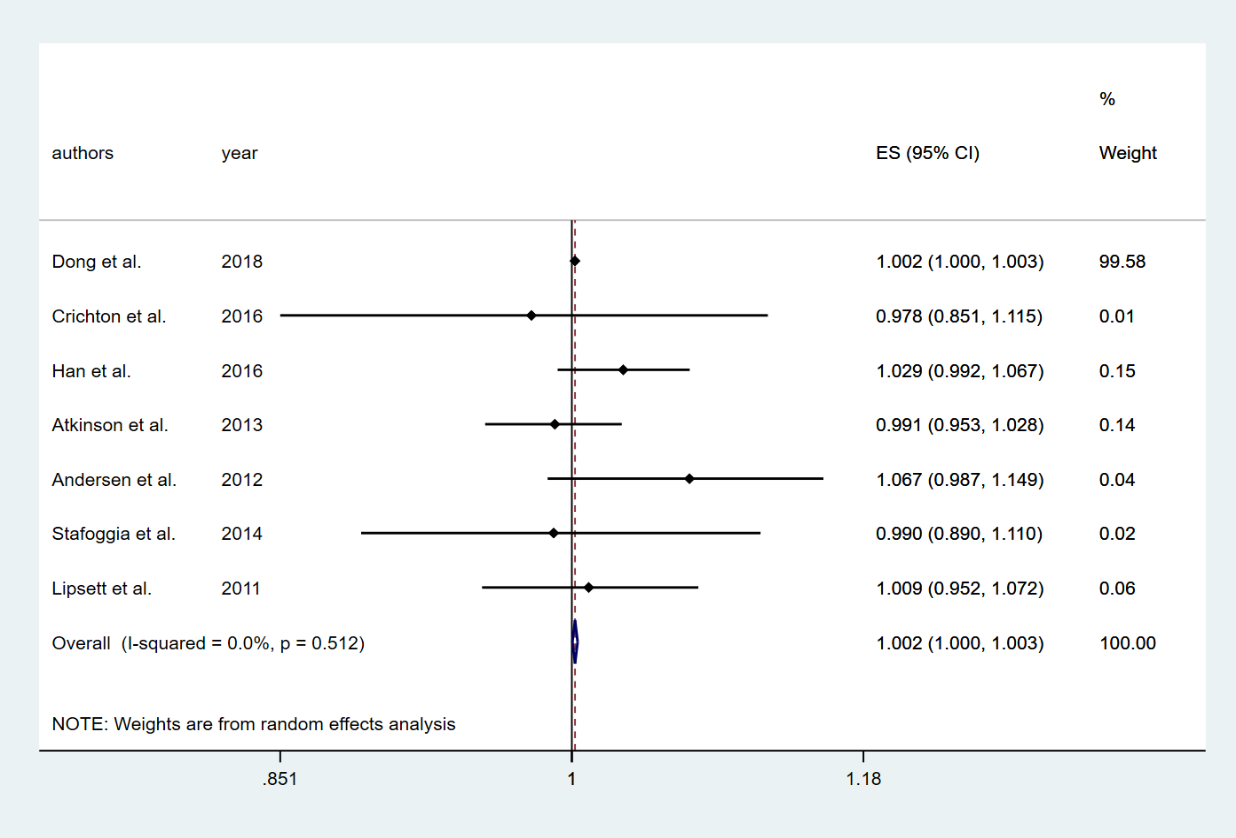


**Figure S10**. Meta-analysis of stroke incidence and exposure to NO_2_ for increments of 10 μg/m^3^.


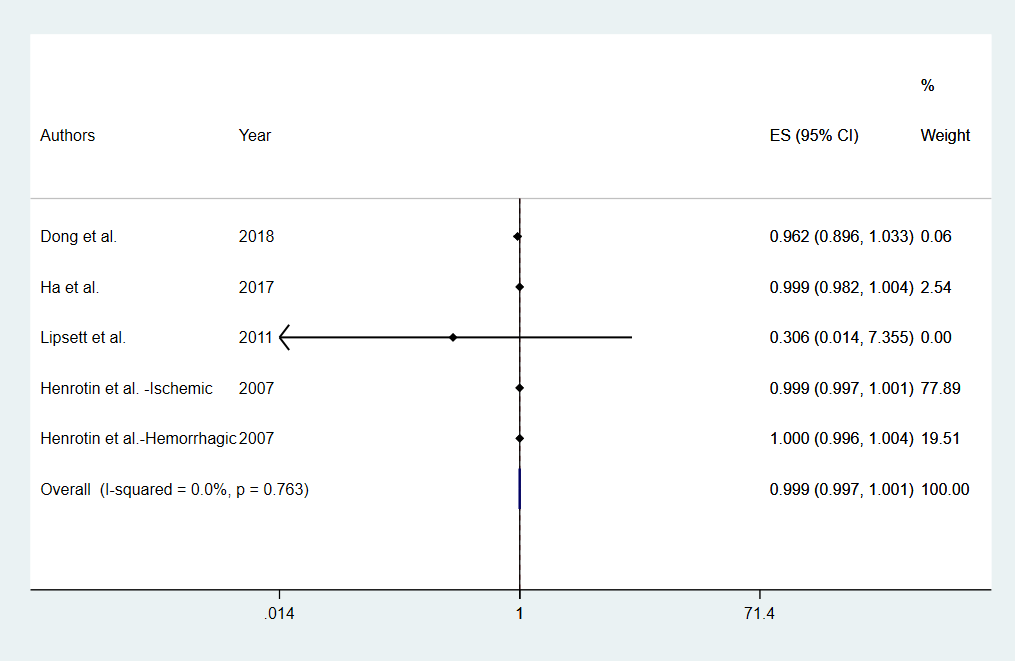


**Figure S11**. Meta-analysis of stroke incidence and exposure to CO for increments of 10 μg/m^3^.


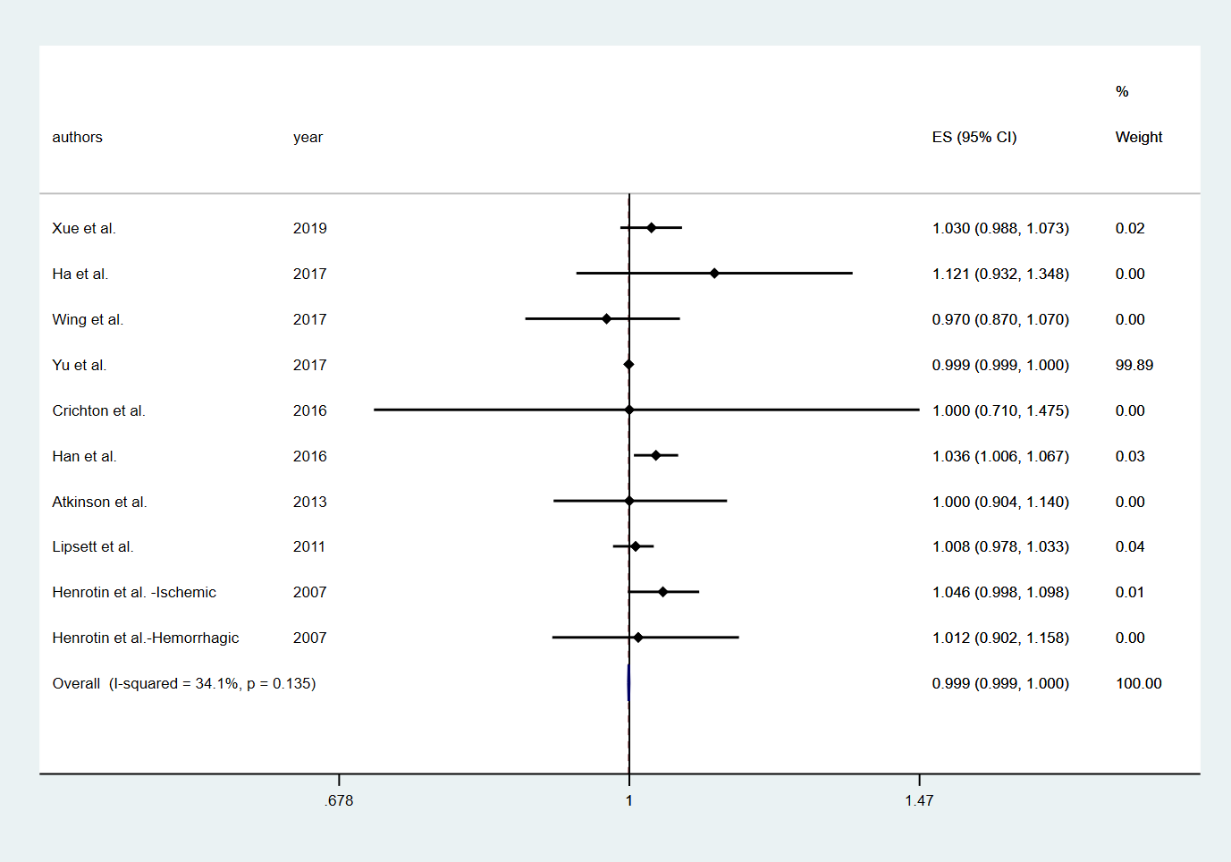


**Figure S12**. Meta-analysis of stroke incidence and exposure to O_3_ for increments of 10 μg/m^3^.


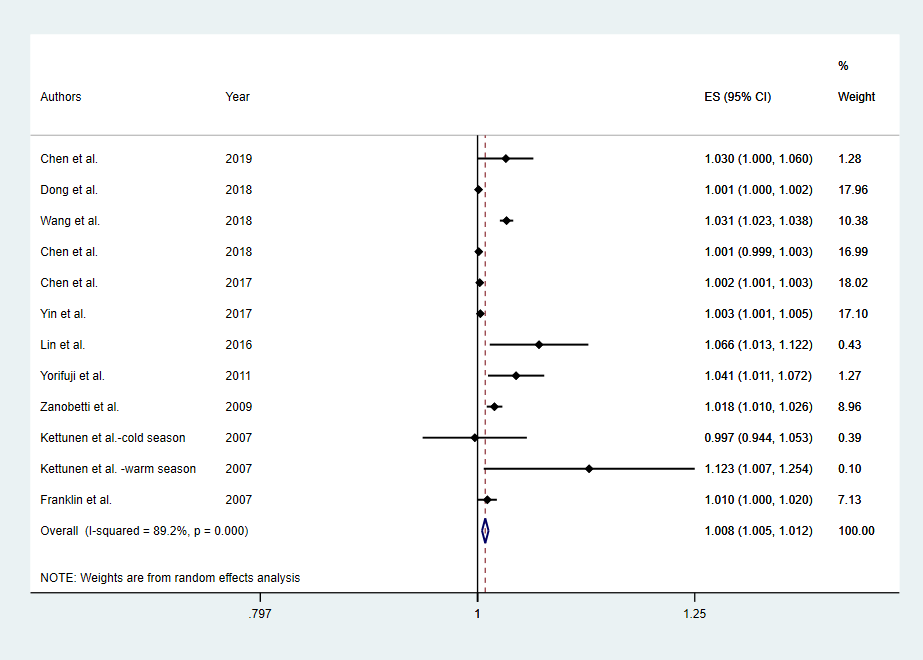


**Figure S13**. Meta-analysis of stroke mortality and exposure to PM_2.5_ for increments of 10 μg/m^3^.


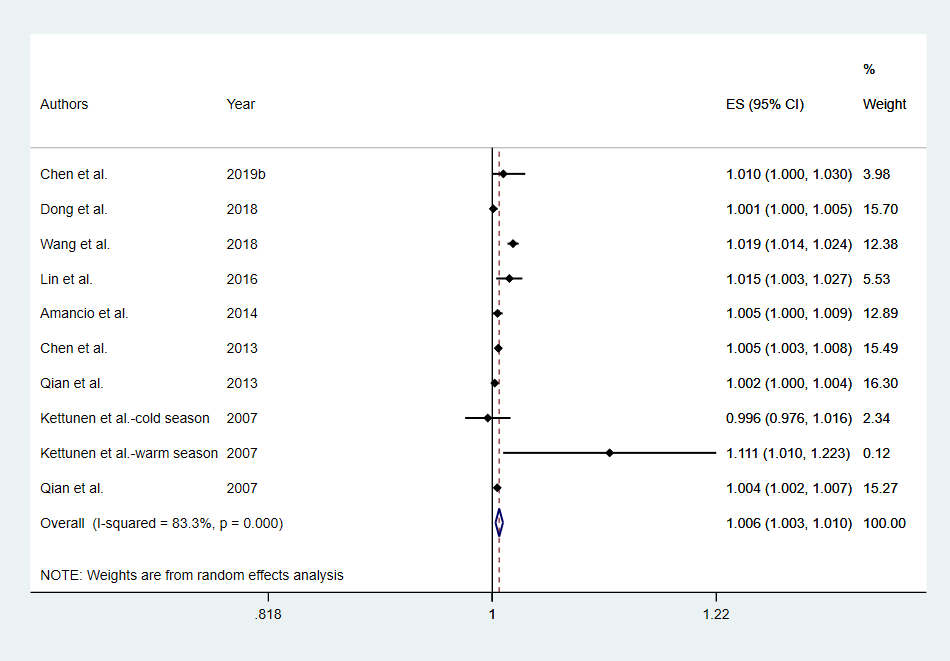


**Figure S14**. Meta-analysis of stroke mortality and exposure to PM_10_ for increments of 10 μg/m^3^.


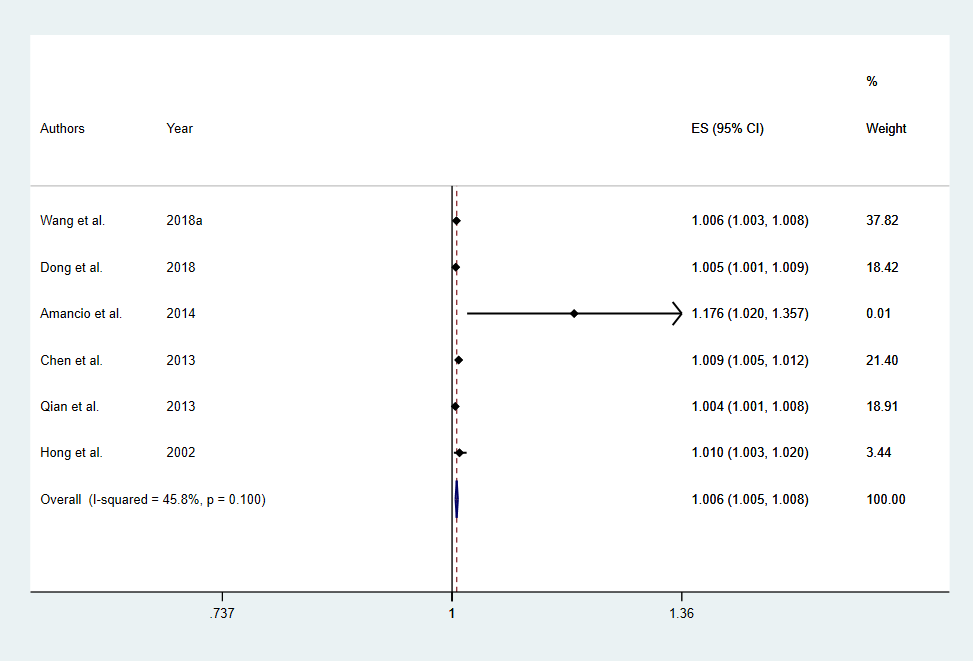


**Figure S15**. Meta-analysis of stroke mortality and exposure to SO_2_ for increments of 10 μg/m^3^.


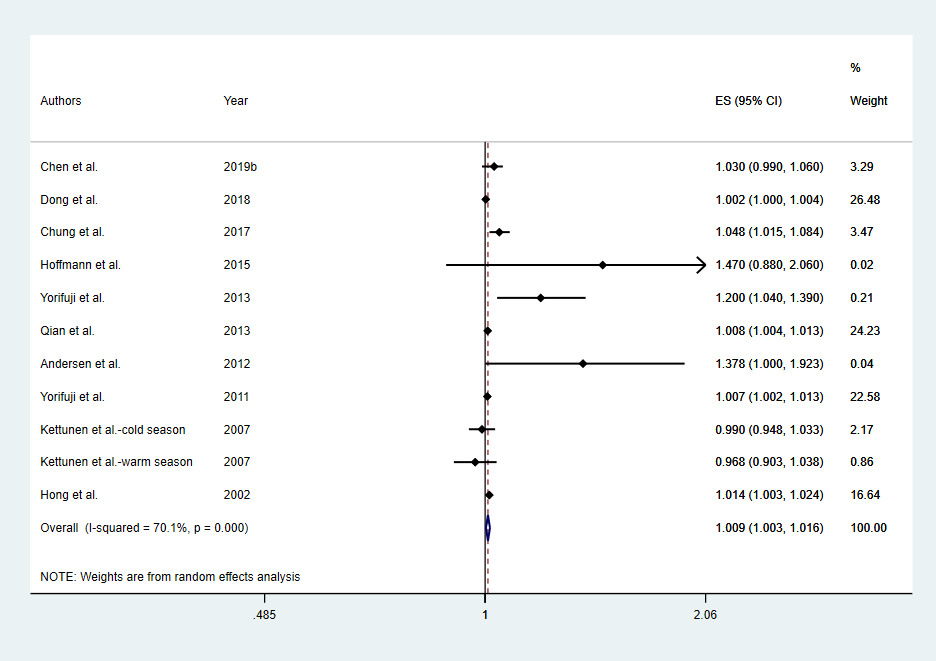


**Figure S16**. Meta-analysis of stroke mortality and exposure to NO_2_ for increments of 10 μg/m^3^.


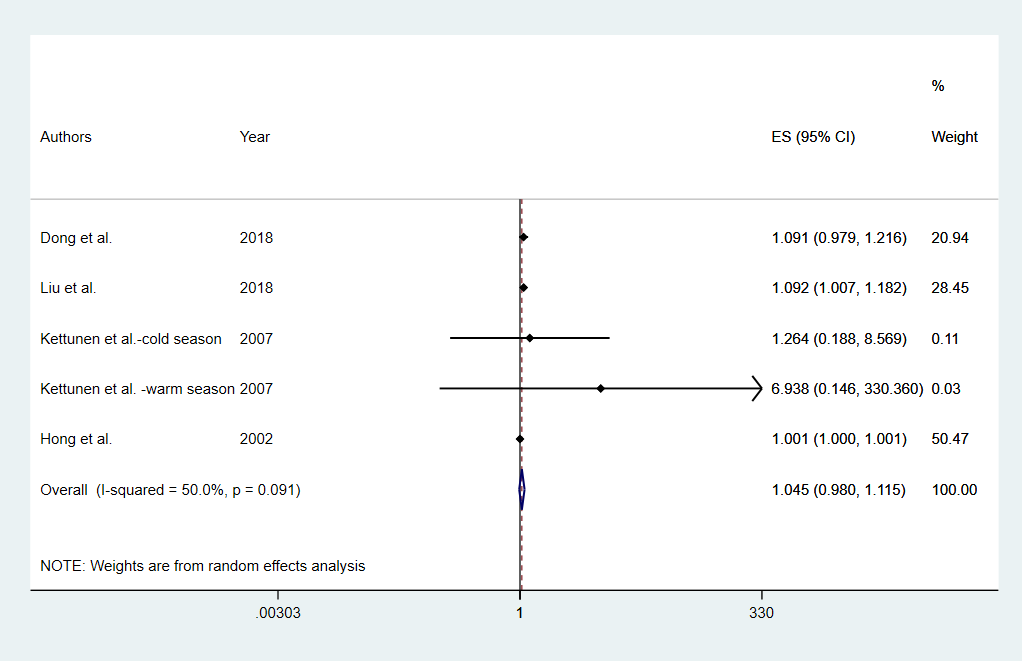


**Figure S17**. Meta-analysis of stroke mortality and exposure to CO for increments of 10 μg/m^3^.


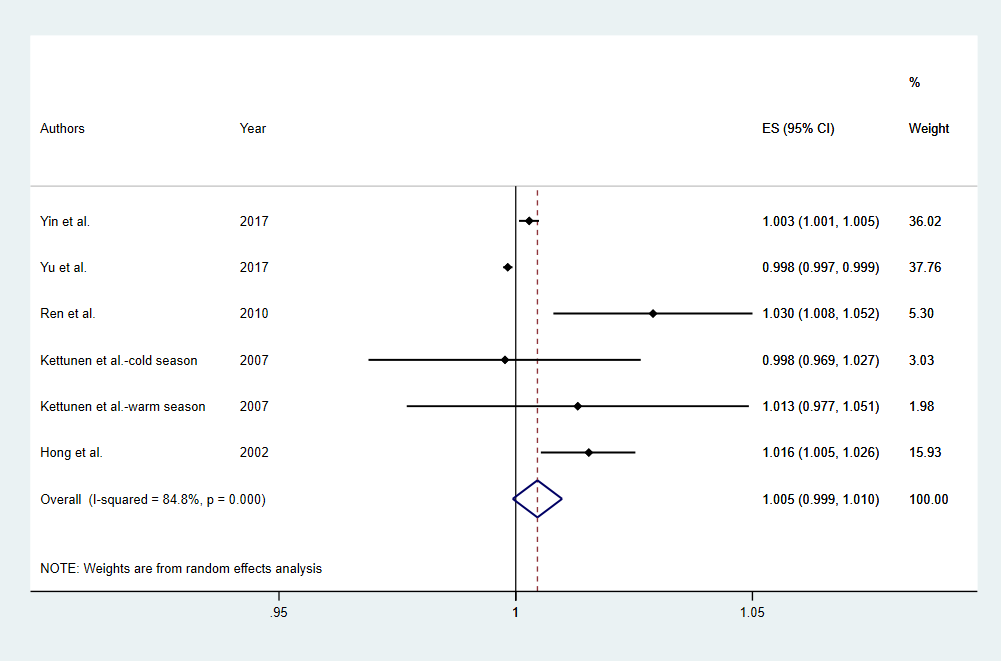


**Figure S18**. Meta-analysis of stroke mortality and exposure to O_3_ for increments of 10 μg/m^3^.

**Figure S19.** Funnel plot analysis on the detection of publication in the meta-analysis of the association between PM_2.5_ and stroke hospital admission

**Figure S20.** Funnel plot analysis on the detection of publication in the meta-analysis of the association between PM_10_ and stroke hospital admission

**Figure S21.** Funnel plot analysis on the detection of publication in the meta-analysis of the association between SO_2_ and stroke hospital admission

**Figure S22.** Funnel plot analysis on the detection of publication in the meta-analysis of the association between NO_2_ and stroke hospital admission

**Figure S23.** Funnel plot analysis on the detection of publication in the meta-analysis of the association between CO and stroke hospital admission

**Figure S24.** Funnel plot analysis on the detection of publication in the meta-analysis of the association between O_3_ and stroke hospital admission

**Figure S25.** Funnel plot analysis on the detection of publication in the meta-analysis of the association between PM_2.5_ and stroke incidence

**Figure S26.** Funnel plot analysis on the detection of publication in the meta-analysis of the association between PM_10_ and stroke incidence

**Figure S27.** Funnel plot analysis on the detection of publication in the meta-analysis of the association between NO_2_ and stroke incidence

**Figure S28.** Funnel plot analysis on the detection of publication in the meta-analysis of the association between CO and stroke incidence

**Figure S29.** Funnel plot analysis on the detection of publication in the meta-analysis of the association between CO and stroke incidence

**Figure S30.** Funnel plot analysis on the detection of publication in the meta-analysis of the association between PM_2.5_ and stroke mortality

**Figure S31.** Funnel plot analysis on the detection of publication in the meta-analysis of the association between PM_10_ and stroke mortality

**Figure S32.** Funnel plot analysis on the detection of publication in the meta-analysis of the association between SO_2_ and stroke mortality

**Figure S33.** Funnel plot analysis on the detection of publication in the meta-analysis of the association between NO_2_ and stroke mortality

**Figure S34.** Funnel plot analysis on the detection of publication in the meta-analysis of the association between O_3_ and stroke mortality

**Figure S35.**Sensitivity analysis of the association between PM_2.5_ and stroke hospital admission by excluding each study

**Figure S36.**Sensitivity analysis of the association between PM_2.5_ and stroke hospital admission by excluding each study

**Figure S37.**Sensitivity analysis of the association between SO_2_ and stroke hospital admission by excluding each study

**Figure S38.**Sensitivity analysis of the association between NO_2_ and stroke hospital admission by excluding each study

**Figure S39.**Sensitivity analysis of the association between CO and stroke hospital admission by excluding each study

**Figure S40.**Sensitivity analysis of the association between O_3_ and stroke hospital admission by excluding each study

**Figure S41.**Sensitivity analysis of the association between PM_2.5_ and stroke incidence by excluding each study

**Figure S42.**Sensitivity analysis of the association between PM_10_ and stroke incidence by excluding each study

**Figure S43.**Sensitivity analysis of the association between NO_2_ and stroke incidence by excluding each study

**Figure S44.**Sensitivity analysis of the association between CO and stroke incidence by excluding each study

**Figure S45.**Sensitivity analysis of the association between O_3_ and stroke incidence by excluding each study

**Figure S46.**Sensitivity analysis of the association between PM_2.5_ and stroke mortality by excluding each study

**Figure S47.**Sensitivity analysis of the association between PM_10_ and stroke mortality by excluding each study.

**Figure S48.**Sensitivity analysis of the association between SO_2_ and stroke mortality by excluding each study.

**Figure S49.**Sensitivity analysis of the association between NO_2_ and stroke mortality by excluding each study.

**Figure S50.**Sensitivity analysis of the association between O_3_ and stroke mortality by excluding each study.
